# Interactions Between Antioxidants: How Useful Are In Vitro Studies for Understanding the Action of Antioxidants in Complex Systems?

**DOI:** 10.3390/ijms27167491

**Published:** 2026-08-21

**Authors:** Izabela Sadowska-Bartosz, Grzegorz Bartosz

**Affiliations:** Laboratory of Analytical Biochemistry, Institute of Food Technology and Nutrition, Faculty of Technology and Life Sciences, University of Rzeszow, 4 Aleksandra Zelwerowicza Street, 35-601 Rzeszow, Poland; gbartosz@ur.edu.pl

**Keywords:** antioxidant interaction, additivity, antagonism, synergy, antioxidant regeneration, ABTS^•^ decolorization, DPPH^•^ decolorization, FRAP, ORAC, Folin-Ciocalteu phenol assay

## Abstract

Interactions between antioxidants are believed to be fundamental for proper nutrition and health benefits. This review discusses various types of antioxidant interactions in vitro, their mechanisms, and their dependence on assay conditions and evaluates the validity of extrapolations of results obtained from cell-free assays and assays of cellular antioxidant activity to in vivo and food systems. In vitro studies revealed that the type of interaction (additive, antagonistic, or synergistic) depends not only on the identity of compounds but also on their absolute concentrations, concentration ratio, type of assay, reaction medium, and the method of analysis of results. Moreover, reactions of antioxidants in simple model systems, employing synthetic indicators, do not fully reflect their reactivity in food systems and organisms. Although in vitro studies may be a useful and necessary step for explaining the mechanisms of interactions between antioxidants, their importance for explaining the phenomena observed in vivo seems limited, as the organismal effects of compounds referred to as antioxidants depend rather on their actions on specific signaling pathways than on their direct antioxidant action. Nevertheless, they may provide useful information for food preservation, allowing for the reduction of the amounts of antioxidant additives.

## 1. Introduction

In recent decades, antioxidants have aroused broad interest in both biomedicine and food science. This interest was partly due to the popularization of the free radical theory of aging, postulating that antioxidants can slow down the aging process [1,2,3], to the beneficial effects of the antioxidant-rich Mediterranean diet [4,5,6], to the occurrence of oxidative stress in many diseases [7,8,9]. In food science, the increasing use of processed food has augmented the demand for safe natural antioxidants [10,11,12].

Studies of individual antioxidants have made it possible to understand the mechanisms of their action at various levels. However, both in organisms and food products, antioxidants are not present individually but coexist with a plethora of other antioxidants, metabolites, proteins, lipids, minerals, and other matrix components. Therefore, interactions among antioxidants are of interest, primarily to understand their action in complex systems.

The null hypothesis concerning the behavior of several antioxidants in a system is that their combined effect is equal to the sum of individual effects (additivity). Such behavior is indeed observed in many systems. However, in other cases, the effect of a mixture of antioxidants is stronger than the sum of the effects of individual antioxidants (synergy) or weaker than the sum of individual components (antagonism). This classification seems intuitively obvious; however, in practice, diagnosis of the type of interaction depends on the mathematical model used to describe the interaction, and distinguishing between additive and non-additive interactions is often more or less arbitrary.

Usually, consuming whole fruits or vegetables shows more beneficial effects than consuming individual dietary supplements, which is widely explained by the interactive effects of coexisting phytochemicals in whole foods [13,14,15]. Interactions between antioxidants may have a practical dimension in food and other systems prone to oxidation, as synergy between antioxidants may reduce the amount of antioxidant additions necessary to prevent oxidation [16,17,18].

An insight into the in vivo interactions between antioxidants is difficult. Therefore, interactions between antioxidants are mainly studied in model in vitro systems, enabling studies of pairs or groups of antioxidants. Can results of such studies be extrapolated to in vivo systems or complex food products? Evaluation of the validity of such extrapolations of results obtained from cell-free assays and assays of cellular antioxidant activity is the aim of the present review.

## 2. Assessment of the Type of Interaction

Antioxidant reactions include electron transfer, hydrogen atom transfer, radical addition, or complexation [19] and can interact with each other. The type of interaction between antioxidants is assessed by comparison of a parameter of an antioxidant assay (e.g., extent of reduction of an indicator, lag time of the reaction) of individual antioxidants and of their mixture. Often, if a linear relationship between the antioxidant activity and the antioxidant concentration is expected, the results of assays are directly compared (direct summation), which is sometimes referred to as the “traditionally calculated mixture effect” [20]. In this approach, if the effect of a mixture of two or more antioxidants coincides (within the limits of experimental error) with the sum of separate effects of components of the mixture, additivity is diagnosed; if the effect of the mixture is higher/lower than the sum of effects of individual components, their interaction is classified as synergy or antagonism, respectively. An important limitation of this approach is the necessity of a strict proportionality between the amounts of antioxidants and the observed effect, which is generally not fulfilled in the ABTS^•^ and DPPH^•^ decolorization, β-carotene bleaching, and ORAC assays. Other models describing interactions imply other criteria of additivity, antagonism, and synergy.

The Chou–Talalay method is one of the most widely used methods for detecting and quantifying synergistic interactions between two or more drugs (for which it was originally developed) or other compounds, including antioxidants. In this approach, the main equation forming the basis of this method was derived as a unified theory of the Michaelis–Menten, Hill, Henderson–Hasselbalch, and Scatchard equations, known as the median-effects equation:

(1) $$\frac{f_{a}}{f_{u}}=\;{\left({\frac{D}{D}}_{m}\right)}^{m}$$

where *f_a_* is the effect (e.g., fraction of an indicator reduced by an antioxidant), *f_u_* is the fraction of indicator unaffected, *D* is the dose of an acting antioxidant, *D_m_* is the median-effect dose, and *m* is the sigmoidicity of the dose–effect curve. This equation can be linearized:

(2) $$log(\frac{f_{a}}{f_{u}})=\;m\;\log(D)\;-\;m\;\log\;(D_{m})$$

From this form of the equation, the values for *D_m_* and _m_ can be estimated. These values can then be used to calculate the effect of the interaction between compounds (antioxidants) using the Combination Index CI:

(3) $$CI=\;\frac{D_{1}}{E_{1}}+\;\frac{D_{2}}{E_{2}}$$

where CI is the effect (e. g. extent of reduction of an indicator) observed for the binary mixture, *D*_1_ and *D*_2_ represent the effect at the single doses of compound A and compound B, and *E*_1_ and *E*_2_ are theoretical doses of the compounds expected to achieve the experimentally measured response. *E*_1_ and *E*_2_ can be calculated using the previously computed *D_m_* and *m* values. CI < 1 indicates synergy, CI = 1 indicates additivity, and CI > 1 indicates antagonism [20,21,22,23,24].

This method also has its limitations. With adequate experimental accuracy of measurements, a minimum of only 10 data points is required for quantitative synergy determination in two-compound combinations [24]. Non-linearity of the dose–response curves, which occurs frequently, may lead to erroneous conclusions on the types of interactions [25].

When the concentration dependence of the reaction of an antioxidant with an indicator is hyperbolic, the method of Webb [26] for the estimation of additivity of effects, derived for enzyme inhibition, can be used:

Expected reduction (fraction) = 1 − [1 − Reduction by Antioxidant 1 (fraction)] × [1 − Reduction by Antioxidant 2 (fraction)](4)

This approach was used, i.a, to estimate interactions between antioxidants in the DPPH^•^ decolorization assay [27] and in the ABTS^•^ decolorization assay [20].

An obvious limitation of all these methods consists in the exactness of matching experimental data with the formula used. If, for any reason, deviation from the expected dependence occurs, the prediction of the combined effect will be charged with error.

A general problem for all methods of evaluation of interactions is the difficulty in distinguishing between additive and non-additive effects when the measured antioxidant activity only slightly deviates from the 100% expected for additivity, or the CI slightly differs from 1. In the “traditionally calculated mixture effect”, a criterion of 10% or even 5% difference [28] from 100% additivity is sometimes arbitrarily used, but usually the statistical significance of differences from 100% additivity is evaluated. As CI is not associated with a standardized statistic or *p* value, designations of significant synergy or significant antagonism must be determined by largely arbitrary thresholds. Chou specifies a narrow range of CI values (0.9–1.1) as “nearly additive” but offers no physical, statistical, or theoretical rationale for those thresholds [29]. More sophisticated statistical methods for determination of confidence intervals have also been proposed [30].

Two examples may illustrate the importance of criteria of differentiation of the type of interaction between antioxidants: the “10% criterion” can lead to different conclusions than statistical evaluation [20,26], and various methods of analysis of interaction can yield divergent conclusions on the types of interactions [20].

Although the statistical evaluation of differences from additivity should be recommended, it is difficult to avoid the impression that the precision of measurements, rather than the real difference in the nature of the interaction, is the discriminating factor in the classification. For this reason, small though statistically significant deviations from additivity are not necessarily an indication of a chemically or biologically meaningful synergy.

## 3. The Type of Interaction Depends on Many Variables

The most common assays used in studies of interactions between antioxidants and their mechanisms, reaction conditions, limitations, and physiological relevance are listed in Table 1.

An extensive review of synergistic, antagonistic, and additive antioxidant interactions in binary antioxidant mixtures in vitro has been published [51], so only exemplary results are reported here (Table 2 and Table 3).

Some unusually high synergistic effects reported for some ORAC combinations are surprising. The authors do not propose an explanation for such strong synergy. It can be suspected that the deviation from the linearity of the assay may be involved, since it is known that ORAC assay is linear only within a range of antioxidant concentrations and, in general, a quadratic regression equation is often used to describe the relationship between the antioxidant concentration and the area under fluorescence curve as a measure of antioxidant activity [41].

Studies of interactions between various antioxidants show that the type of interaction depends on many variables, as discussed below.

### 3.1. The Type of Interaction Depends on the Assay

The same mixture of compounds can show different types of interaction when assessed by different assays. A mixture of ascorbic acid, chlorogenic acid, and cysteine showed additivity in superoxide scavenging, antagonism in scavenging of the hydroxyl radical, and synergy in the scavenging of *tert*–butyl peroxide radical in an EPR assay [60]. Interaction of curcumin with (−)-epicatechin or with the epicatechin fraction from green tea showed synergy in the ABTS^•^ decolorization assay but antagonism in the inhibition of lipid peroxidation. In the ABTS^•^ decolorization assay, the strongest effect was observed with the equimolar mixture of pure compounds [28].

The mechanisms of various assays are different (Table 1), which may be one reason for the discrepancies in results on the interactions of antioxidants. However, comparison of interactions of S-allyl-L-cysteine with a range of phenolics showed different types of interactions (synergy vs. antagonism) in two assays (ABTS^•^ decolorization and DPPH^•^ decolorization) acting via the same mechanism (SPLET) [64] (Table 2).

Even within one method, classification of the type of interaction may depend on the method of prediction of the additive effect. A study of interactions between flavonoids (taxifolin, quercetin, rutin, and morin) and glutathione (GSH) found weak antagonistic effects (90–97% of the expected additive effect) when using the “traditional” method of analysis based on the sum of results for individual compounds and mostly weak synergistic effects (99–111% of the additive effect) when analyzing the data using Webb’s simulation. In the first mode of analysis, a weak concentration effect was observed in the taxifolin-GSH and rutin-GSH systems, the interaction effect decreasing with the decrease in the flavonoid/GSH ratio [20]. Authors of this study also compared the ABTS^•^ decolorization assay with another assay employing ABTS, in which lag time caused by antioxidants was measured after the addition of potassium persulfate to the solution of ABTS. In this method, strong synergistic effects were observed in the systems of taxifolin-GSH, quercetin-GSH, and rutin-GSH, while a weak antagonistic effect or no significant effects were found in the morin-GSH system. The authors concluded that the manifestation of the synergy strongly depends on the mode of inflow of the free radicals.

In studies of the interactions of various antioxidants with red cabbage extract, synergy was found for glutathione in the FRAP assay but not in the ABTS^•^ decolorization and ORAC, and for Trolox in the ORAC assay but not in ABTS^•^ decolorization and FRAP [78,79].

The reactivity of antioxidants for various indicators used in the antioxidant assays differs [80], and results may depend on the specific interactions with indicators rather than on interactions between antioxidants. Therefore, the use of more than one analytical approach should be recommended for the assessment of antioxidant synergy/antagonism.

### 3.2. The Type of Interaction Depends on the Identity of Interacting Compounds

The dependence of interactions on the classes of interacting compounds seems obvious, but even within the defined groups of similar compounds, the type of interaction varies for different compounds. In the DPPH^•^ decolorization assay of antioxidant activity, equimolar mixtures of gallic and protocatechuic acid, of gallic acid and chlorogenic acid, and of protocatechuic acid and chlorogenic acid showed higher antioxidant activity than sums of activities of individual components (synergistic interaction), while the antioxidant activity of a mixture of gallic acid and vanillic acid was lower than the sum of activities of individual components (antagonistic interaction) [52].

In the ORAC assay employing 0.625 µM final antioxidant concentrations in the reaction mixture, the ORAC values for a combination of two hydroxybenzoic acids were mainly synergistic and ranged from 70% (gallic acid + vanillic acid) to 336% (gentisic acid + syringic acid) of the value expected for additive interactions. Interactions between three (from 102 to 148% of the values expected for additivity) and four hydroxybenzoic acids (139–167% of the values expected for additivity) were mostly or totally synergistic [28].

Among combinations of two hydroxycinnamic acids, the interactions were mainly synergistic (from 98% of the expected value for *p*-coumaric acid + rosmarinic acid) to 411% of the expected value (*p*-coumaric acid + ferulic acid). Interactions between three hydroxycinnamic acids were mainly antagonistic (78–116% of respective values predicted for additivity) and interactions between four acids were antagonistic, additive, or synergistic (86–144% of the values predicted for additivity) [28].

There was no synergistic effect in the interactions between the selected phenolics (catechin, chlorogenic acid, cyanidin, cyanidin 3-glucoside, cyanidin 3-rutinoside, epicatechin, peonidin, peonidin 3-glucoside, quercetin, quercetin 3-glucoside, quercetin 3-galactoside, and quercetin 3-rutinoside) in the ABTS^•^ decolorization assay, and only additive effects were observed, although in some cases the effects were apparently more than additive (but the deviations from the additivity did not exceed several percent), and in some cases antagonistic effects occurred [81].

A photochemical electrode based on a g-C_3_N_4_/NiS/TiO_2_ sensor demonstrated synergistic interactions in various binary mixtures between resveratrol, catechinic acid, epicatechin, proanthocyanidins, pyrogallol, ascorbic acid, and gallic acid, as well as in complex mixtures composed of five or six components. However, an antagonistic interaction was noted for a system of proanthocyanidins/gallic acid [75].

Catechin significantly decreased the rate of decolorization of malvidin 3-glucoside and peonidin 3-glucoside but not delphinidin 3-glucoside, petunidin 3-glucoside, or cyanidin 3-glucoside during AAPH-induced peroxidation of linoleic acid and a considerably higher prolonging effect on the lag time of oxygen consumption was observed for malvidin 3-glucoside and peonidin 3-glucoside than for three other anthocyanins [82].

In a study of antioxidant interaction in the off-odor formation during oxidation of linoleic acid, the type of interaction changed from synergy to additivity and even to antagonism depending on the aldehyde product analyzed [76].

### 3.3. The Type of Interaction Depends on the Absolute Concentrations and the Concentration Ratio of Interacting Compounds

In studies of interactions between hydroxybenzoic acids (protocatechuic, gentisic, gallic, vanillic, and syringic acids) at a 3.2 µM concentration (final concentration in the reaction mixture) in the FRAP assay, all mixtures containing gentisic acid showed a synergistic effect (128–189% of the values expected for additivity). The mixture of protocatechuic and syringic acid showed an additive effect, while all others exerted an antagonistic effect (down to 42% of the values expected for additivity). At a concentration of 16.1 µM, a synergistic effect was observed only for the mixture of gentisic + syringic acids, while at 32.3 µM, the synergistic effect disappeared. Among ternary mixtures, the mixture of protocatechuic + gentisic + syringic acids showed the greatest synergistic effect (274% of the value expected for additivity at the 3.2 µM concentration), but this effect decreased at the concentration of 16.1% (107% of the expected value) and vanished at the concentration of 32.3 µM (99% of the expected value). Most of the ternary combinations of hydroxycinnamic acids showed antagonistic interactions; one of the few exceptions was the mixture of *p*-coumaric acid, caffeic acid, and rosmarinic acid, showing synergistic interactions (227% of the value expected for additivity at 3.2 µM concentration, 148% of the expected value at 16.1 µM concentration, and 111% of the expected value at 32.3 µM concentration). For combinations of four hydroxycinnamic acids, antagonistic interactions predominated at the concentrations of 3.2 and 16.1 µM, with additive or weakly synergistic interactions at 32.3 µM [52].

A FRAP study of the interactions between flavonoids in binary systems at various proportions of components, from 3:1 to 1:3, showed again the dependence of the effects on the antioxidant concentration ratio. In the catechin + quercetin and catechin + quercetin-3-β-glucoside systems, synergy was observed; the magnitude of the synergistic effect increased with a decreasing proportion of catechin (from 28% to 37% and from 33% to 51%, respectively). However, no such regularity was observed in other binary systems [71].

In the DPPH^•^ decolorization assay, quercetin showed a synergistic interaction with resveratrol in a molar ratio of 1:1, 2:1, and 3:1, but antagonistic interaction in ratios of 1:2 and 1:3. Hydroquinone showed a synergistic effect with kaempferol over a molar ratio of 1:3–3:1, with a maximal effect at the 2:1 ratio. The interaction of hydroquinone with resveratrol was weakly synergistic over the concentration range of 1:2 to 3:1. The interaction of kaempferol with resveratrol was weakly antagonistic over a ratio of 1:3. In contrast, the synergistic effects of interactions of rutin with resveratrol increased progressively with the increase in the rutin/resveratrol molar ratio in the range of 1:3 to 3:1, and the effect of quercetin/resveratrol rose progressively from antagonism (quercetin/resveratrol molar ratio of 1:3 and 1:2) to synergy at increasing quercetin/resveratrol ratios, up to 3:1 [66].

In a study of interactions between trehalose and ascorbic acid, catechin, gallic acid, and quercetin, antagonistic interactions were observed, with the interaction coefficient decreasing as antioxidant concentrations decreased [67]. However, interpretation of these results is not straightforward since trehalose did not reduce DPPH^•^ at all, so the effect cannot be referred to as an interaction between antioxidants, but was rather conditioned by other mechanism(s).

Binary antioxidant formulations: *tert*–butyl hydroxyquinone (TBHQ):butyl-4- hydroxytoluene (BHA), *tert*–butyl hydroxyquinone (TBHQ):propyl gallate, and TBHQ: pyrogallol showed the best synergistic effect at 2:1, 1:1, 2:1 weight ratios, respectively, in both distilled soybean-oil- and distilled poultry-fat-based biodiesel [83].

### 3.4. The Type of Interaction Depends on the Reaction Medium

The type of interaction between antioxidants obviously depends on the reaction medium. Viscosity affects the reaction rates of antioxidants [84]. A change in pH and decrease in medium polarity affect the ionization state and solubility, partitioning between phases; thus, interactions between antioxidants promote aggregation of less polar antioxidants. Redox potentials of the reagents, reaction kinetics, and predominant antioxidant mechanisms may all change with pH. The appearance of amphiphilic structures leads to partitioning of more hydrophobic antioxidants, which changes their local concentrations and their interactions, with hydrophilic antioxidants remaining in the aqueous phase, complicating reaction kinetics with respect to a homogeneous system [85,86,87,88]. A “polar paradox” observed in such systems consists in a tendency to concentrate non-polar antioxidants in the lipid environment, so if the oxidation process takes place in the hydrophobic phase, the apparent activity of non-polar antioxidants is higher than that of polar antioxidants [89]. Micellar charge will also affect the distribution of ionic antioxidants [90].

α-Tocopherol and ϒ-oryzanol showed mostly antagonistic interactions in rice bran oil [69] but a synergistic interaction in refined coconut oil [70]. It supports the view that the same combination of minor constituents may show diverse antioxidant interactions in different lipid environments.

The interaction between resveratrol and a tocopherol analogue, 2,2,5,7,8-pentamethyl-6-chromanol (PMHC), in the prevention of oxidation of methyl linoleate was additive in Triton X-100 [58] and in SDS micelles but synergistic in CTAB micelles [91] and a liposomal system. In DMPC liposomes, it depended on pH in the range of 4–8, with maximal synergy observed at pH 7. This effect was attributed to the pH dependence of the localization of resveratrol within the lipid region, affecting the probability of reaction of this compound with the tocopheroxyl radical [58]. The interaction of resveratrol with PMHC in the inhibition of lipid peroxidation with PMHC was additive in SDS micelles, and synergy was observed in CTAB micelles [92]. Further examples of the dependencies shown above can be found in Table 2 and Table 3.

The dependence of antioxidant interaction on the medium is very important and should be considered in attempts to extrapolate results of in vitro experiments to in vivo situations or food systems.

## 4. Mechanisms of Interactions Between Antioxidants

Several mechanisms have been proposed to account for the non-additivity of antioxidant interactions in vitro; first of all, the regeneration of antioxidants.

### 4.1. Regeneration of Antioxidants

In an assay based on a reduction of a free radical of an indicator like DPPH^•^ or ABTS^•^ decolorization assay, an antioxidant A reacts with the free radical (e.g., ABTS^•^), reducing it to the non-radical indicator molecule (ABTS) and forming the antioxidant free radical A^•^

A + ABTS^•^ → A^•^ + ABTS (5)

The antioxidant free radical can react with a second radical of the indicator, reducing it and forming a two-electron oxidation product of the antioxidant A_ox_

A^•^ + ABTS^•^ → A_ox_ + ABTS (6)

Alternatively, A^•^ can be reduced by another antioxidant, forming a free radical of another antioxidant B, regenerating the initial, reduced form of the antioxidant [93,94]:

A^•^ + B →A + B^•^ (7)

In the ORAC assay, the situation is somewhat more complicated: antioxidants can react with peroxyl radicals, preventing oxidation of the indicator (usually fluorescein), or regenerate the indicator from its radical formed upon oxidation by peroxyl or alkoxyl radicals [40,41,95].

Most often, the synergistic interaction between antioxidants has been attributed to regeneration of the antioxidant radical or fully oxidized antioxidant formed in the assay by another antioxidant of lower redox potential. The classic example is the regeneration of tocopherol from the tocopheryl radical by ascorbic acid [96,97]. Ascorbic acid was postulated to regenerate the tocopheryl radical formed in reactions of tocopherol with peroxyl radicals of lipids undergoing peroxidation. This regeneration is thermodynamically possible, taking into account the standard redox potentials of the tocopheryl radical/tocopherol redox pair (500 mV) and semidehydroascorbyl radical/ascorbate anion redox pair (282 mV) [98]. In a homogeneous medium containing both tocopherol and ascorbate, ascorbate was utilized first [97]. In a heterogenous system, this synergism may be even more important. α-Tocopherol is lipid-soluble, but its hydroxyl group and phytol chain give it amphiphilic properties. Therefore, α-tocopherol partitions at the interface of cell membranes to protect against lipid oxidation. When free radicals are present within the membrane, α-tocopherol is oxidized to form a more polar α-tocopheroxyl radical, which can interact with water-soluble ascorbic acid at the membrane surface and initiate the electron transfer required to regenerate α-tocopherol back to its original form.

ROO^•^ + Tocopherol → ROOH + Tocopherol^•^ (8)

Tocopherol^•^ + Ascorbate^−^ → Tocopherol + Ascorbate^•−^ (9)

where ROO^•^ is a lipid peroxyl radical and ROOH is lipid hydroperoxide [97].

The ascorbate radical is relatively stable and can disproportionate, forming ascorbate and dehydroascorbate [99].

Ascorbate^•−^ + Ascorbate^•−^ → Ascorbate^−^ + Dehydroascorbate^−^ (10)

In the egg phosphatidylcholine–dicetylphosphatidylcholine liposome system, lipid peroxidation was significantly retarded in the presence of tocopherol + ascorbate compared with tocopherol alone [100]. Regeneration of tocopherol was also observed in membrane models and in biological membranes [101]. Glutathione did not affect the protective effect of tocopherol significantly [100].

The property to regenerate tocopherol from its radical was postulated to be shared by other compounds. Ubiquinol and phenolic compounds such as epigallocatechin gallate (EGCG), gallic acid, and epicatechin were also reported to regenerate α-tocopherol from its radical [102,103]:

Ubiquinol + Tocopherol^•^ → Ubiquinol^•^ + Tocopherol (11)

Phenolic + Tocopherol^•^ → Phenolic^•^ + Tocopherol (12)

A synergistic interaction was observed in a system of alpha-tocopherol + green tea catechins (epigallocatechin gallate, epigallocatechin, or epicatechin gallate (ECG)) + ascorbic acid in the inhibition of linoleic acid in SDS micelles. While tocopherol and catechins induced a lag period, ascorbic acid alone did not delay the onset of substrate oxidation. In the system containing tocopherol, a catechin, and ascorbic acid, ascorbic acid was consumed first, followed by catechin and tocopherol as the last compound. Addition of ascorbic acid to ECG solution changed the EPR signal of the ECG radical into the signal of the ascorbyl radical, pointing to the regeneration of the alpha-tocopherol radical as the mechanism for the synergistic action of catechins and ascorbate [59].

Autoxidation of several model substrates (stripped sunflower oil, squalene, and styrene) was inhibited by PMHC and caffeic acid phenethyl ester (CAPE). With all substrates, γ-terpinene acted synergistically. The combination of either PMHC or CAPE with γ–terpinene did not significantly change the slope of the inhibited period of styrene oxidation compared to that produced by each of the phenols alone, but it extended its duration in a dose-dependent fashion while not retarding the onset of styrene oxidation itself [74]. Similar results were obtained in studies of oxidation of stripped sunflower oil at 130 °C, where γ–terpinene prolonged the antioxidant activity of α-tocopherol and PMHC [104].

The synergy of γ-terpinene with PMHC or CAPE was ascribed to the regeneration of the chain-breaking antioxidants PMHC and CAPE from their radicals to original catechols [74]. Similarly, pyrogallol, being the more effective antioxidant, readily donates a hydrogen atom from its hydroxyl group to fatty acid free radicals, thereby forming an antioxidant radical. TBHQ then transfers hydrogen to the antioxidant radical to regenerate it back to pyrogallol

ROO^•^ + Pyrogallol → ROOH + Pyrogallol^•^ (13)

Pyrogallol^•^ + TBHQ → Pyrogallol + TBHQ^•^ (14)

In the process, TBHQ is converted to a radical that can form stable products with other free radicals, and this effect, together with the interaction and regeneration of pyrogallol, represents an effective synergistic effect between the two antioxidants. Analysis of distilled soybean-oil-based biodiesel stored for 3 months with 2:1 TBHQ:pyrogallol showed the total amount of pyrogallol close to its original value [83].

The synergism between β-carotene and astaxanthin or between lycopene and astaxanthin, and the lack of synergism between zeaxanthin and astaxanthin in the inhibition of lipid peroxidation induced by the hydrophobic initiator AMVN, has been explained by the regeneration of astaxanthin at the expense of β-carotene or lycopene. According to this proposal, astaxanthin, located at the lipid–water interface, acts as a radical bridge channeling the electron from the more reducing carotenoid in the inner part of the membrane to the interface, where the radicals with high dipole moment are concentrated. Zeaxanthin and astaxanthin do not act synergistically, as they both have similar spatial distribution in the membrane [56].

ROO^•^ + Astaxanthin → ROOH + Astaxanthin^•^ (15)

Astaxanthin^•^ + Lycopene → Astaxanthin + Lycopene^•^ (16)

Resveratrol was reported to be neither an outstanding antioxidant nor a co-antioxidant in homogeneous solutions [105] at the lipid–water interface. Resveratrol scavenges LOO^•^ within the liposomal membrane; thus, resveratrol belongs to the same class of lipophilic antioxidants as α-tocopherol [106].

The mere regeneration of one antioxidant by another does not change the amount of free radicals in the system and cannot explain the synergy of antioxidant action. However, it can lead to the effect of synergy, taking into account the broader context of antioxidant reactivity.

**Figure 1:**
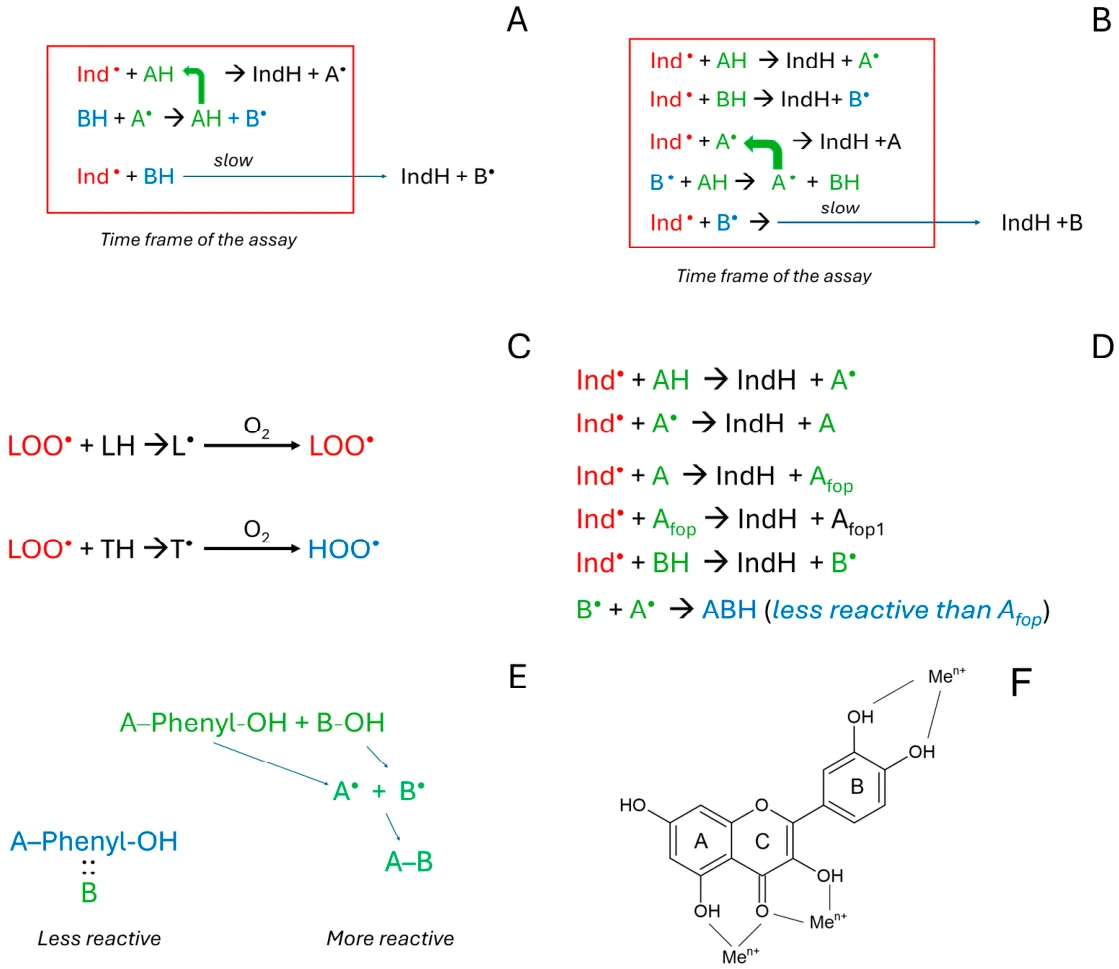
Mechanisms of non-additive interactions between antioxidants (presented in the literature and hypothetical). Regeneration of antioxidant A from its radical may increase the antioxidant activity within the reaction time frame if another antioxidant B reacts with the indicator (Ind) at a slower rate (**A**) or if antioxidant A regenerates B from its radical that has low reactivity for the indicator (**B**). Ind^•^, free radical indicator, e.g., ABTS^•^ or DPPH^•^; IndH, reduced indicator. (**C**), Change of reaction mechanism: antioxidants such as γ-terpinene (T) change the propagating radical from LOO^•^ to HOO^•^, more susceptible to termination [107]. (**D**) Formation of an adduct AB, less reactive than the still-reactive further oxidation products A_fop_ of A [83]. (**E**) Non-covalent or covalent interactions with another antioxidant may decrease or increase the reactivity of antioxidants [60]. (**F**) Metal chelation may affect the reactivity of antioxidants; e.g., flavonoids [108,109]. Red, oxidant; Green, antioxidant; Blue, less reactive oxidant or antioxidant.

(i) The second antioxidant is not reactive with the probe but reacts with A^•^. This may seem improbable since the thermodynamic condition for A to react with the free radical indicator is that the redox potential of the redox system (A^•^/A) must be lower than the redox potential of the redox system (indicator radical/reduced indicator). To be able to regenerate A, the redox potential of the (B^•^/B) system must be lower than the redox potential of the (A^•^/A) system. If so, why should it not reduce the indicator radical itself? In principle, it should, but apart from the thermodynamic condition, kinetic factors (e.g., caused by a steric obstacle) may cause such a reaction to be slow or impossible [80]. If the reaction is slow, it may not be completed within the limited time of the assay. Regeneration of the more reactive antioxidant A allows it to reduce more indicator molecules within the time frame of the assay (Figure 1A).
(ii) The free radical of an antioxidant such as Trolox may react with the free radical of an indicator. However, for other antioxidants, this radical may not be as reactive as the native antioxidant. In such a case, regeneration of A from A^•^ will increase the reactivity of A. This mechanism will be important if antioxidant B is less reactive for the indicator than A; otherwise, it would hardly affect the overall activity of antioxidants in the system (Figure 1B).

The mechanism (ii) has been considered in an attempt to explain the differences in the extent of cooperation of catechin with different anthocyanins. The spin densities on the phenolic O atoms estimated by a semiempirical quantum chemical method differed for various anthocyanins. The radicals generated by delphinidin 3-glucoside, petunidin 3-glucoside, and cyanidin 3-glucoside (first class), having an *o*-dihydroxy structure on the B ring, show a low spin density (3–4%) on the phenolic O atoms, while those generated by malvidin 3-glucoside, peonidin 3-glucoside, and pelargonidin 3-glucoside (second class) are characterized by a spin density on phenolic O atoms of the order of 24% [82]:

ROO^•^ + Anthocyanin → ROOH + Anthocyanin^•^

Anthocyanin^•^ + Catechin → Anthocyanin + Catechin^•^

However, in both cases, considering reactions in a real system, the synergy found in an in vitro assay may represent an experimental artefact since, over a longer time, the slower antioxidant would have a chance to react as well, especially when exposed to a continuous flux of reactive oxygen species (ROS).

### 4.2. Change of Reaction Mechanism

One antioxidant can change the reaction mechanism in a way favorable for another antioxidant. Another effect was proposed to explain the synergistic effect of γ-terpinene in the peroxidation of lipids. According to this proposal, terpinene changes the propagation chain-carrier from the peroxyl radicals ROO^•^ to hydroperoxyl radicals HOO^•^ (Figure 1C). γ-Terpinene is rapidly attacked by ROO^•^ and releases HOO^•^ [104].

γ-Terpinene + ROO^•^ → γ-Terpinene^•^ + ROOH

γ-Terpinene^•^ + O_2_ → HOO^•^ + *p*-Cymene

Hydroperoxyl radicals can both propagate the oxidation and be quenched by another HOO^•^ or by ROO^•^ (self-termination or cross-termination). Since the self-termination of HOO^•^ and its cross-termination with ROO^•^ is much faster than the self-termination of ROO^•^, the overall termination efficiency would increase in the presence of γ-terpinene [104,107].

### 4.3. Formation of Antioxidant Hetero- and Homodimers, and Other Reactive Products

Generally, the set of reactions occurring during assays of antioxidant activity is much broader than those symbolized in Equations (5)–(7). They may include recombination between antioxidant radicals and further reactions of oxidized antioxidants. For example, GSH is oxidized to glutathione disulfide (GSSG), but GSSG can still react with ABTS^•^ to form higher oxidation products [20,32]. Reaction of the GSH radical with a radical of another antioxidant leads to the formation of a glutathione conjugate. If the conjugate is less reactive than GSSG, this reaction will contribute to an antagonistic interaction between both antioxidants (Figure 1D); if it is more reactive, it may lead to a synergistic interaction. Analogous situations can occur with other pairs of antioxidants. Some authors classify such interactions as “apparent synergy” [110].

**Figure 2:**
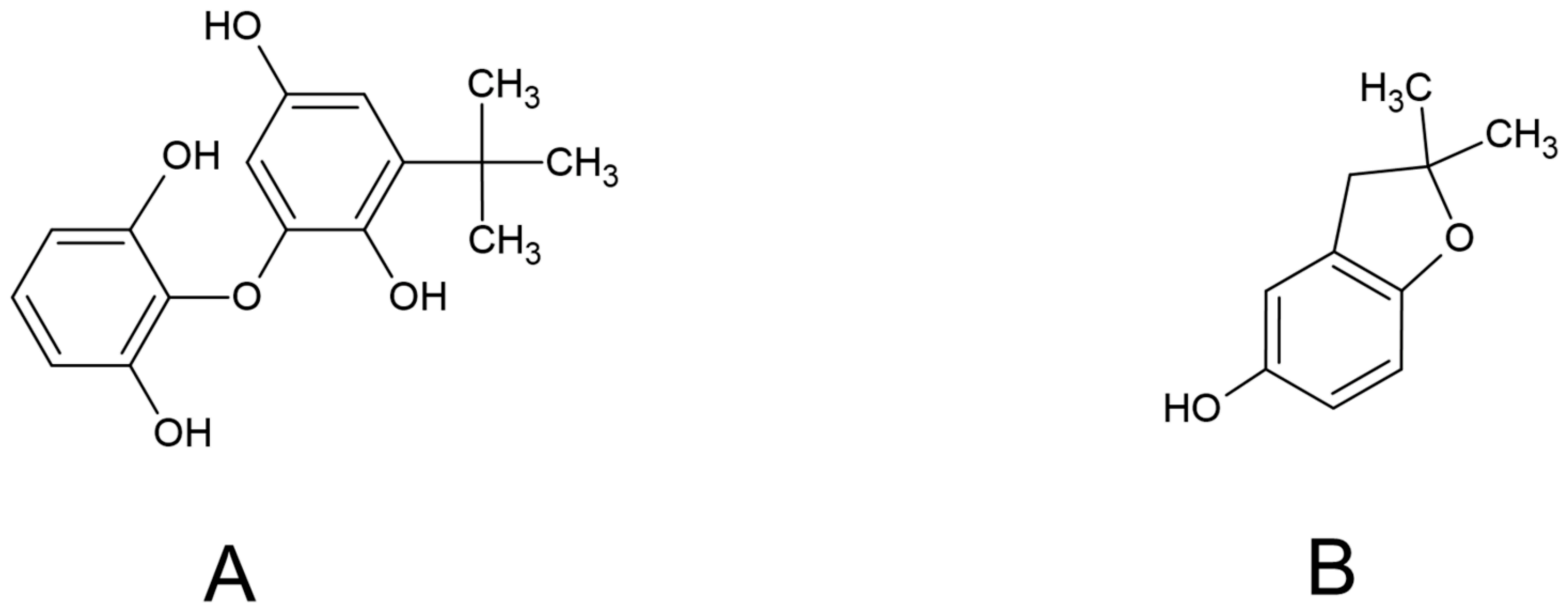
A heterodimer formed in the reaction of TBHQ and pyrogallol [83] (**A**), and 2,2-dimethyl-2,3-dihydro-benzo(b)furan, a reactive product formed during oxidation of TBHQ (**B**) [111].

Formation of dimers between antioxidant radicals may produce new antioxidant species that contain two phenols, which in effect are stronger or weaker antioxidants than the parent antioxidants (Figure 1E). Examples of such dimers that are stronger antioxidants are shown in Figure 2.

Flavonoids lacking the catechol group in the B ring showed antagonistic behavior with GSH. Myricetin displayed an additive effect, while quercetin, fisetin, luteolin, luteolin-7-O-glucoside, taxifolin, and (+)-catechin demonstrated synergistic actions. Quinones formed by oxidation of phenol groups may react with GSH, forming adducts [112], which may affect DPPH^•^ scavenging. Among the flavonoids tested, those having a catechol group in the B ring, namely quercetin, fisetin, luteolin, luteolin-7-O-glucoside, taxifolin, and (+)-catechin, showed synergistic effects with glutathione. An exception was noted with quercetagetin, which has one additional hydroxyl group at C6 of the A ring compared to quercetin. Adducts formed at C2′ and C5′ of the B ring seem to be more important for the DPPH^•^ reduction than adducts formed at C6 and C8 of the A ring [27].

Antioxidant homodimers, higher oligomers, or further degradation products may also be formed, and if they retain the reactivity with the oxidant (or if their reactivity is even enhanced), this effect may contribute to synergy between antioxidants when another antioxidant facilitates their formation (e.g., when a dimer-forming antioxidant regenerates another antioxidant). Resveratrol radicals formed in reaction with peroxyl radicals during lipid peroxidation may form dimers and further cyclization products, with recovered hydroxyl groups able to trap peroxyl radicals. The formation of such dimers is facilitated in micellar and liposomal systems due to the increased concentration of resveratrol at the lipid–water interface at pH 6–7 and may be responsible for increased antioxidant activity and synergistic effects of resveratrol [106]. A benzo(b)furan derivative formed during oxidation of TBHQ, 2,2-dimethyl-2,3-dihydro-benzo(b)furan, also shows a strong antioxidant activity [111]. Products of melatonin oxidation retain the antioxidant activity, forming a “radical scavenging cascade” [113,114]. Products of the reaction of chrysin with ABTS^•^ have an even higher reactivity for ABTS^•^ than the parent compound [115].

Sugars may cause a reduction in the antioxidant activity of the polyphenols. Condensation reactions can occur between sugar molecules and polyphenols; in consequence, glycosides like pentagalloylglucose and tetragalloylglucose are likely to be formed. However, sucrose molecules can also interact with oxidized phenolic compounds; as a result, reduced forms of phenolic may be formed, resulting in an increase in antioxidant activity [116].

### 4.4. Non-Covalent Interactions Between Antioxidants

Formation of intermolecular hydrogen bonds between antioxidant molecules may be an important factor. While compounds that are non-hydrogen-bonded (free) are expected to possess full activity in electron or hydrogen atom transfer mechanisms, this activity may be diminished by hydrogen bonding and modified by other non-covalent interactions [117,118,119]. When both carboxyl groups and phenolic hydroxyl groups are present in one system, possible bonding could occur between the two kinds of groups, which may account for the antagonistic interactions of gallic acid with proanthocyanidins [75]. H-bonding between flavonoids may result in a decrease in the availability of -OH groups and thus a decrease in the antioxidant activity [60].

### 4.5. Metal Chelation

Many antioxidants, including flavonoids, have the ability to chelate metal ions, which catalyze various oxidation reactions but may also have antioxidant properties in their lower oxidation states. Metal ions, especially ferrous or ferric ions, may be present as trace contaminants of reagents, e.g., buffer components or as components of the sample studied [120,121]. Antioxidants may bind these ions, reduce ferric ions, or both bind and reduce ions at higher oxidation states. Binding and reduction of metal ions may diminish the intrinsic antioxidant activity of organic compounds, but metal ions may increase the activity of the complex in comparison with the parent compound (Figure 1F). Both these effects can contribute to the hypo- or hyperadditivity of antioxidant mixtures [17]. Complexation of flavanols with metal ions mainly decreased, but in some cases increased their reactivity in the ABTS^•^ decolorization assay [108]. Metal complexes of flavonoids such as quercetin, rutin, galangin, and catechin had better activity in the DPPH^•^ decolorization assay than the parent flavonoids [109]. It could be expected that metal chelation can especially affect the FRAP assay, based on Fe(III) reduction. However, Fe(III) is present in high excess over antioxidants in this assay, and minute amounts that may be present in the analyzed sample do not interfere with the assay [37].

## 5. Interactions of Antioxidants with Proteins

Antioxidants may interact with proteins in food and with blood plasma proteins, forming non-covalent complexes or covalent conjugates, which may affect antioxidant reactivity and the antioxidant activities of proteins [17,122,123,124,125,126]. Complexation with proteins may protect the antioxidant activity of flavonoids in the digestive tract [125]. Often, such interactions enhance the antioxidant activity of proteins but decrease the antioxidant activity of small-molecular-weight antioxidants, although exceptions to this rule have been reported [127]. Binding of a small-molecular-weight antioxidant may induce conformational changes of a protein. Studies of the interactions of EGCG and caffeic acid with whey proteins demonstrated both synergistic and antagonistic effects. The former were explained by protein conformational changes that allow free radicals to access nucleophilic centers previously inaccessible because of steric hindrance, while the latter were ascribed to H-bond formation between protein groups and the hydroxyl groups of phenolic compounds, thereby reducing the number of available hydroxyl groups and altering the ability to donate electrons [125,127,128,129]. Digestion of protein complexes with small-molecular-weight antioxidants can lead to a release of the latter from the complexes. Formation of complexes between rice proteins and phenolic acids increased the antioxidant activity of the proteins but decreased their digestibility [130]. Chlorogenic acid showed synergy with wheat proteins and casein, especially after simulated digestion. It has also been hypothesized that polyphenol–protein systems can clear free radicals by forming more stable reaction products, thereby terminating the free radical chain reaction [126].

## 6. Interactions Between Antioxidants in the Folin–Ciocalteu Phenol Assay

The Folin–Ciocalteu assay for phenolic compounds is also a redox reaction of a complicated mechanism [131,132].

In the assay of total phenolics employing the Folin–Ciocalteu reagent, additivity was observed in the combination of extracts of different spices and pure phenolics with synthetic antioxidants, although in some cases a synergistic effect was evident [133]. When studying legume/non-legume/fruit food combinations, mostly antagonistic interactions were observed [134]. For mixtures of green tea (*Camellia sinensis*), honey, and lemon (*Citrus limonum*) extract, synergistic effects were found (144.0–181.3% of values expected for additivity) [135].

In the mixtures of spirulina (*Arthrospira*) with apple (*Malus domestica*) juice and with Japanese quince (*Chaenomeles japonica*) syrup, the measured phenolic content was 77% and 74%, respectively, of the sum of those measured for single components [136].

Such observation casts doubt on the validity of the assay; due to interactions between the components of analyzed samples, its results may not reflect the real contents of these compounds.

## 7. Antioxidant Interactions in Complex Food Products

Antioxidant interactions between food products and extracts have also been the subject of numerous studies. Of course, in these studies, the mechanisms underlying these interactions are more difficult, if possible at all, to assess.

A synergistic interaction of green tea with honey and lemon extract was demonstrated in the ABTS^•^ decolorization assay [134]. Synergistic interactions were found between sweet potato (*Ipomoea batatas*) extracts, tea polyphenols, and kudzu vine (*Pueraria*) flavonoids by DPPH^•^ decolorization and FRAP assays [137].

Extracts of leaves of ivy gourd (*Coccinia grandis*) and sambong (*Blumea balsamifera*) showed both synergistic and antagonistic interactions in the ABTS^•^ decolorization assay, depending on the ratio of the extracts, and mostly synergistic interactions in the DPPH^•^ decolorization assay [138].

Antagonistic or synergistic interactions between ethanolic extracts of leaves of mango (*Mangifera indica*) and the aerial parts of asthma weed (*Euphorbia hirta*) were observed, depending on the ratio of the extracts, in the FRAP assay [139].

Interactions between herbal components of a TC-16 polyherbal formulation comprising turmeric (*Curcuma longa*), Bentong ginger (*Zingiber officinale* var. Bentong), black pepper (*Piper nigrum*), and calamondin (*Citrofortunella macrocarpa*) with giant honeybee (*Apis dorsata*) honey were antagonistic in the ABTS^•^ decolorization, DPPH^•^ decolorization, and FRAP assays and synergistic in ORAC and beta-carotene bleaching assays [140].

In the DPPH^•^ decolorization assay, strongly synergistic interactions were revealed between methanolic extracts of edible portions of khejri desert legumes, khejri (*Prosopis cineraria*) and gum Arabic tree (*Acacia senegal*), non-legumes (fragrant manjack *Cordia dichotoma* and karira *Capparis decidua*), and mango (experimental EC_50_ values < expected EC_50_ values). In the FRAP assay, mostly synergistic but also additive and antagonistic interactions were found [134].

Interaction of spirulina with apple juice was additive in the FRAP assay and antagonistic in the ABTS^•^ decolorization assay; interaction of spirulina and Japanese quince syrup was synergistic in the FRAP assay and additive in the ABTS^•^ decolorization assay; interaction between spirulina and cranberry (*Vaccinium macrocarpon*) syrup was synergistic in the FRAP assay and antagonistic in the ABTS^•^ decolorization assay [136]. The latter study also demonstrated considerable differences between interactions in initial food products and their bioavailable fractions.

Interactions between essential oils of bay laurel (*Laurus nobilis*), Spanish lavender (*Lavandula stoechas*), and pennyroyal (*Mentha pulegium*) were mainly synergistic but additive or antagonistic in some cases, depending on the proportions of the oils and method of assay (ABTS^•^ decolorization, DPPH^•^ decolorization and CUPRAC) [141].

Mostly synergistic effects were found in the interaction between extracts of peel of bitter orange (*Citrus aurantium*) and aerial parts of field wormwood (*Artemisia campestris*) and white wormwood (*Artemisia herba alba*) in the ABTS^•^ decolorization and FRAP assays [142].

Analysis of interactions between various food products using the QUENCHER procedure [143] and ABTS^•^ decolorization assay demonstrated antagonistic, additive, and synergistic interactions between various products and, often, changes in the type of interaction after simulated gastric, intestinal, and colonic digestion (exemplary results shown in Table 4 and Table 5). Some of the observed antagonistic interactions can be due to the binding of polyphenols to milk proteins [144].

On the basis of analysis of synergy, Liu et al. conclude that esculetin and gentisic acid may be the principal phenols to enhance the oxidation stability of tea seed oil [70].

## 8. Cellular Antioxidant Activity

Cellular antioxidant activity, based on estimation of inhibition of oxidation of intracellular fluorescent probe upon treatment with an oxidant of cells preincubated with antioxidants [48,49], is the next step from in vitro cell-free assays towards a situation in the cell. Moreover, they allow study of the effects of antioxidants on other cellular functions, apart from the level of reactive oxygen species. However, they have serious limitations, too. The scavenging of ROS responsible for inhibition of oxidation of fluorescent probes is contributed to by endogenous intracellular antioxidants and antioxidant enzymes. The transport of antioxidants to the cells may affect their intracellular concentrations, which are, in fact, unknown. Exogenous antioxidants may be subject to intracellular metabolism. All these factors lead to differences in studies of the same antioxidants obtained with various cell types.

In rat cardiomyocyte H9c2 cells, the interactions between delphinidin and lycopene, chlorogenic acid and lutein, chlorogenic acid and lycopene, delphinidin and chlorogenic acid, delphinidin and lutein, and lycopene and lutein ranged from synergistic to antagonistic in all cases, depending on the concentration ratio of antioxidants, as demonstrated by cellular activity assay measuring the inhibition of ROS formation [24].

In the cellular antioxidant activity assay using human umbilical vein endothelial cells (HUVECs), Caco-2 human colorectal adenocarcinoma cells, and L-02 cells, the observed effects were cell-line dependent. The interactions between lutein and quercetin were antagonistic, additive, or synergistic in HUVECs and Caco-2 cells and antagonistic or additive in L-02 cells, depending on the concentration ratio of the components. The interactions between lutein and luteolin were synergistic in HUVECs and antagonistic, additive, or synergistic in Caco-2 and L-02 cells. Lycopene and quercetin showed synergy in HUVECs and Caco-2 cells and synergy, additivity, or antagonism in L-02 cells. The interactions between lycopene and luteolin were synergistic in HUVECs, antagonistic or synergistic in Caco-2 cells, and antagonistic, additive, or synergistic in L-02 cells. These differences between cell lines were attributed mainly to the effect of carotenoids on the expression of membrane transporters able to mediate flavonoid uptake by the cells [62].

## 9. Discussion

In vitro studies are believed to be a necessary initial step for elucidation of the mechanisms of interactions between antioxidants before moving on to in vivo studies. However, the results of these studies should be interpreted with caution. The reaction conditions used in most assays (solvent, pH, temperature, and the absence of a natural matrix and other compounds, such as metal ion chelators) may differ from the physiological conditions of antioxidant action, thereby limiting their physiological relevance (Table 1); e.g., the DPPH^•^ decolorization assay usually employs methanol or ethanol as the reaction medium [34,35,36], and the FRAP assay is run at a pH of 3.6 [37,38], which is rather far from the physiological pH range. The time frame of the assays is limited, so slow reactions may not be included. Moreover, the oxidants/probes in some antioxidant assays (ABTS^•^, DPPH^•^, fluorescein in ORAC) are synthetic radicals not occurring in nature, and reduction of these radicals by antioxidants does not necessarily correlate with protection of biologically relevant substrates such as DNA, proteins, or lipids.

In cellular antioxidant activity assays, the concentrations of antioxidants used are usually much higher than those to which cells can be exposed in the organism, and the actual intracellular concentrations of antioxidants during the assay are unknown. One point of criticism is that most studies of antioxidant interactions in vitro are performed using the “black box” approach, not including analysis of reaction products, and not aiming at elucidation of the mechanisms of antioxidant reactions and interactions.

However, the main problem concerns the relevance of these studies to the question of organismal effects of compounds referred to as antioxidants. Endogenous antioxidants and antioxidant vitamins interact with others, but the antioxidant system involves many compounds, much more than those studied in simple in vitro systems. Some of them cooperate with enzymes, being oxidized and regenerated in enzymatic reactions, not included in in vitro experiments.

Other exogenous plant-derived antioxidants (mainly phenolics) are secondary plant metabolites, synthesized for defense against environmental stress [146,147], herbivores [148,149,150] and microorganisms [151,152].

The physiological effects of these compounds are complex. One is the direct antioxidant action. However, most exogenous antioxidants are generally absorbed in minute amounts [153,154,155]. Such amounts of exogenous antioxidants generally cannot significantly affect the redox equilibrium of the human body [156]. Moreover, they are subject to metabolic transformations by gut microbiota and the host detoxification system and elimination. Flavonoid metabolites have different biological and antioxidant properties than their parent compounds, suggesting that data from in vitro studies using nonmetabolized flavonoids are of limited relevance for their in vivo effects [157]. Nevertheless, they can be locally accumulated in higher amounts and affect cellular and tissue redox signaling [158]. NF-κB is a ubiquitous transcription factor that regulates many central events in normal cell function and fate. NF-κB is redox-sensitive, and, in general, oxidants promote its activation while antioxidants inhibit it. Flavanols and procyanidins can interfere with NF-κB activation not only by altering cellular redox state, but also by specific binding to proteins involved in the NF-κB pathway. (−)-Epicatechin and dimers can inhibit NADPH oxidase and the subsequent superoxide production by directly binding to the enzyme [159].

Apart from their chemical reactions with oxidants, antioxidants exert biological effects mediated through signaling pathways and changes in gene expression. (−)-Epicatechin and procyanidins were demonstrated to directly inhibit protein phosphatases, kinases, or other signaling proteins [159]. *Pueraria* flavonoids induce autophagy via stimulation of the PI3K/Akt/mTOR signaling pathway [160], the PI3K/Akt/HIF-1α signaling pathway to mitigate cancer resistance [161], and the Wnt/β-catenin pathway, important for cancer growth and metastasis [162] and inhibiting the cGAS-STING signaling pathway ameliorating LPS-induced acute lung injury [163], to mention only a few examples.

In biological membranes, flavonoids interfere with the raft structure and thus membrane signaling [164]. Naringenin inhibits acid sphingomyelinase-mediated membrane raft clustering and reduces NADPH oxidase activation in this way [165]. EGCG affects lipid rafts, which blocks the activation of the c-Met receptor and the HGF/c-Met pathway responsible for invasion and metastasis of most human cancers [166].

These effects may be related to or independent of the antioxidant activity of polyphenols. However, interaction with signaling pathways may mediate the antioxidant effects of these compounds at the cellular and organismal levels. There are many reports on the activation of the Keap1-Nrf2 pathway, the primary cellular defense system against oxidative stress, activating genes responsible for the synthesis of antioxidant proteins and mounting endogenous antioxidants (e.g., [167,168,169,170]).

These effects, more important than the direct antioxidant action of phenolics [171,172], escape detection by in vitro assays of antioxidant activity and antioxidant interactions.

## 10. Conclusions and Perspectives

Taking all above aspects into account, the importance of results of studies of interactions between antioxidants for understanding the in vivo effects of antioxidants seems limited.

However, in vitro studies of antioxidant interactions may be useful for food preservation. Studies of the interactions of antioxidants in the prevention of the oxidation of oils and other fats can be directly translated into practical protocols, allowing for limiting the amounts of antioxidant additives. However, antioxidant interactions characterized in model systems should be validated in the actual food matrix under realistic processing and storage conditions.

Future studies on the in vitro interactions of antioxidants should include thorough analysis of multiple concentrations and ratios, concentration–response dependencies, kinetic analysis, careful selection of predefined additivity models, inclusion of mechanistically distinct assays, and appropriate statistics. They should aim at elucidation of the molecular mechanisms of the interactions, combining antioxidant assays with analytical techniques, enabling identification of intermediates, final products, and byproducts of apparently simple antioxidant assays. They should use substrates and conditions relevant to physiology or food systems. Finally, they require validation in relevant food or biological systems. Understanding the real course and pathways of antioxidant reactions should allow explaining and predicting interactions between antioxidants, also operating in vivo.

## Figures and Tables

**Table 1:** Mechanisms, conditions, limitations, and physiological relevance of main assays used in studies of antioxidant interactions.

Assay (Mechanism)	Oxidant/Indicator	Medium	pH	Major Limitations	Physiological Relevance	References
ABTS^•^ decolorization (SPLET)	ABTS^•^	Aqueous or ethanol	Usually 7.4 but may be lower	Limited reactivity of hydrophobic antioxidants; short reaction times underestimate the activity of slow-reacting antioxidants	Limited (non-physiological substrate)	[31,32,33]
DPPH^•^ decolorization (SPLET)	DPPH^•^	Methanol or ethanol	Usually not controlled	Limited solubility of some antioxidants; insolubility of proteins. Colored compound may interfere	Limited (non-physiological substrate)	[34,35,36]
FRAP (SET)	Fe(III)	Acetate buffer	3.6	Low reactivity of thiols. Colored compound may interfere	Limited (unphysiological pH, Fe(III) is not a major physiological oxidant)	[37,38]
ORAC (HAT)	Usually APPH/fluorescein	Phosphate buffer	Usually 7.0–7.4	Needs a fluorimeter or fluorimetry reader; time-consuming	Physiologically relevant (measures reactivity for peroxyl radicals, measurement usually at 37 °C)	[39,40,41]
β-Carotene bleaching (HAT)	Spontaneous or AAPH, metal ions, or heme/Carotene	Aqueous buffer + detergent	Variable, usually 7.0–7.4	Various protocols, time-consuming	Physiologically relevant (measures reactivity for peroxyl radicals)	[42,43,44]
Inhibition of oxidation of linoleic acid, oils or other fats (HAT, RAF)	Spontaneous or AAPH, metal ions, or heme/O_2_ uptake, conjugated dienes or Rancimat	Aqueous buffer + detergent	Variable, usually 7.0–7.4	Various protocols, time-consuming	Physiologically relevant (measures reactivity for peroxyl radicals)	[45,46,47]
Cellular antioxidant activity	Usually AAPH/fluorogenic ROS-sensitive probes	Cells pre-loaded with antioxidants	Usually 7.4	Interference from endogenous antioxidants, intracellular concentration of exogenous antioxidants unknown; cell-type dependence	Physiologically relevant (measures antioxidant activity in cells)	[48,49,50]

HAT, hydrogen electron transfer; RAF, radical adduct formation; SET, single electron transfer; SPLET, sequential proton loss electron transfer.

**Table 2:** Exemplary quantitative results of interactions between antioxidants in vitro.

Antioxidant System	Assay	Interaction Parameter = 100% × [(Effect Measured)/(Effect Predicted as the Arithmetic Sum of Individual Antioxidant Activities)]; Type of Interaction		Reference
3.8 µM Taxifolin + 4.2 µM GSH	ABTS^•^ decolorization, “classical” analysis	88.9	Ant ^T^	[20]
1.8 µM Taxifolin + 9.4 µM GSH	ABTS^•^ decolorization, “classical” analysis	90.1	Add ^T^	[20]
1.2 µM Taxifolin + 11.9 µM GSH	ABTS^•^ decolorization, “classical” analysis	91.3	Ant ^T^	[20]
0.7 µM Taxifolin + 11.1 µM GSH	ABTS^•^ decolorization, “classical” analysis	91.5	Ant ^T^	[20]
3.8 µM Taxifolin + 4.2 µM GSH	ABTS^•^ decolorization, Webb’s simulation	102.9	Add ^T^	[20]
1.8 µM Taxifolin + 9.4 µM GSH	ABTS^•^ decolorization, Webb’s simulation	102.9	Add ^T^	[20]
1.2 µM Taxifolin + 11.9 µM GSH	ABTS^•^ decolorization, Webb’s simulation	101.5	Add ^T^	[20]
0.7 µM Taxifolin + 11.1 µM GSH	ABTS^•^ decolorization, Webb’s simulation	97.8	Add ^T^	[20]
2.3 µM Quercetin + 2.6 µM GSH	ABTS^•^ decolorization, “classical” analysis	97.1	Add ^T^	[20]
1.3 µM Quercetin + 6.5 µM GSH	ABTS^•^ decolorization, “classical” analysis	92.9	Ant ^T^	[20]
0.8 µM Quercetin + 7.7 µM GSH	ABTS^•^ decolorization, “classical” analysis	94.4	Add ^T^	[20]
0.7 µM Quercetin + 10 µM GSH	ABTS^•^ decolorization, “classical” analysis	89.5	Add ^T^	[20]
2.3 µM Quercetin + 2.6 µM GSH	ABTS^•^ decolorization, Webb’s simulation	110.6	Syn ^T^	[20]
1.3 µM Quercetin + 6.5 µM GSH	ABTS^•^ decolorization, Webb’s simulation	104.8	Add ^T^	[20]
0.8 µM Quercetin + 7.7 µM GSH	ABTS^•^ decolorization, Webb’s simulation	103.1	Add ^T^	[20]
0.7 µM Quercetin + 10 µM GSH	ABTS^•^ decolorization, Webb’s simulation	97.7	Add ^T^	[20]
0.1 mM Gallic acid + 0.1 mM procatechuic acid	DPPH^•^ decolorization	141.5	Syn ^A^	[21]
0.1 mM Gallic acid + 0.1 mM chlorogenic acid	DPPH^•^ decolorization	102.1 ^#^	Syn ^A^	[52]
0.1 mM Protocatechuic acid + 0.1 mM chlorogenic acid	DPPH^•^ decolorization	112.9 ^#^	Syn ^A^	[52]
0.1 mM Gallic acid + 0.1 mM vanillic acid	DPPH^•^ decolorization	91.7 ^#^	Ant ^A^	[52]
0.1 mM Protocatechuic acid + 0.1 mM vanillic acid	DPPH^•^ decolorization	103.5 ^#^	Add ^A^	[52]
66 µM Gallic acid + 66 µM chlorogenic acid + 66 µM vanillic acid	DPPH^•^ decolorization	177.6 ^#A^	Syn ^A^	[52]
66 µM Gallic acid + 66 µM procatechuic acid + 66 µM chlorogenic acid	DPPH^•^ decolorization	106.3 ^#A^	Syn ^A^	[52]
66 µM Gallic acid + 66 µM procatechuic acid + 66 µM vanillic acid	DPPH^•^ decolorization	120.8 ^#A^	Syn ^A^	[52]
66 µM Protocatechuic acid + 66 µM chlorogenic acid + 66 µM vanillic acid	DPPH^•^ decolorization	82.0 ^#A^	Ant ^A^	[52]
Caffeic acid + α-tocopherol:	DPPH^•^ decolorization			[53]
0.2:1		144	Syn ^N10^	
0.5:1		159	Syn ^N10^	
1:1		133	Syn ^N10^	
2:1		138	Syn ^N10^	
Quercetin + α-tocopherol:	DPPH^•^ decolorization			[53]
0.5:1		180	Syn ^N10^	
1:1		118	Syn ^N10^	
2:1		116	Syn ^N10^	
5:1		163	Syn ^N10^	
2.94 µM Gallic acid + 11.8 µM caffeic acid	FRAP	237.8	Syn ^N10^	[16]
2.94 µM Rosmarinic acid + 11.8 µM caffeic acid	FRAP	137.5	Syn ^N10^	[16]
2.94 µM Quercetin + 2.94 µM rutin	FRAP	106.3	Add ^N10^	[16]
2.94 µM Quercetin + 2.94 µM rutin + 11.8 µM caffeic acid	FRAP	159.4	Syn ^N10^	[16]
2.94 µM Quercetin + 2.94 µM rutin + 2.94 µM rosmarinic acid	FRAP	155.2	Syn ^N10^	[16]
3.2 µM *p*-Coumaric acid + 3.2 µM sinapic acid	FRAP	96.0	Add ^N5^	[28]
16.1 µM *p*-Coumaric acid + 16.1 µM sinapic acid	FRAP	108.0 ^#^	Syn ^N5^	[28]
32.3 µM *p*-Coumaric acid + 32.3 µM sinapic acid	FRAP	118.0 ^#^	Syn ^N5^	[28]
3.2 µM Caffeic acid + 3.2 µM rosmarinic acid	FRAP	96.2 ^#^	Add ^N5^	[28]
16.1 µM Caffeic acid + 16.1 µM rosmarinic acid	FRAP	112.1 ^#^	Syn ^N5^	[28]
32.3 µM Caffeic acid + 32.3 µM rosmarinic acid	FRAP	115.8 ^#^	Syn ^N5^	[28]
3.2 µM *p*-Coumaric acid + 3.2 µM caffeic acid + 3.2 µM rosmarinic acid	FRAP	226.6 ^#^	Syn ^N5^	[28]
16.1 µM *p*-Coumaric acid + 16.1 µM caffeic acid + 16.1 µM rosmarinic acid	FRAP	147.6 ^#^	Syn ^N5^	[28]
32.3 µM *p*-Coumaric acid + 32.3 µM caffeic acid + 32.3 µM rosmarinic acid	FRAP	110.8 ^#^	Syn ^N5^	[28]
0.625 µM *p*-Coumaric acid + 0.625 µM ferulic acid	ORAC	410.3 ^#^	Syn ^N5^	[28]
0.625 µM Caffeic acid + 0.625 µM sinapic acid	ORAC	311.5 ^#^	Syn ^N5^	[28]
0.625 µM Gentisic acid + 0.625 µM syringic acid	ORAC	336.2 ^#^	Syn ^N5^	[28]
0.625 µM Vanillic acid + 0.625 µM syringic acid	ORAC	310.4 ^#^	Syn ^N5^	[28]
0.625 µM Protocatechuic acid + 0.625 µM gentisic acid + 0.625 µM vanillic acid	ORAC	149.1 ^#^	Syn ^N5^	[28]
0.625 µM Protocatechuic acid + 0.625 µM gentisic acid + 0.625 µM vanillic acid + 0.625 µM syringic acid	ORAC	165.4 ^#^	Syn ^N5^	[28]
0.625 µM Ferulic acid + 0.625 µM sinapic acid + 0.625 µM rosmarinic acid	ORAC	77.0 ^#^	Ant ^N5^	[28]
0.625 µM Rosmarinic acid + 0.625 µM *p*-coumaric acid + 0625 µM ferulic acid + 0.625 µM sinapic acid	ORAC	142.6 ^#^	Syn ^N5^	[28]
0.625 µM Protocatechuic acid + 0.625 µM gentisic acid + 0.625 µM gallic acid 0.625 µM vanillic acid + 0.625 µM syringic acid	ORAC	127.7 ^#^	Syn ^N5^	[28]
0.625 µM *p*-Coumaric acid + 0.625 µM caffeic acid + 0.625 µM ferulic acid 0.625 µM sinapic acid + 0.625 µM rosmarinic acid	ORAC	101.8 ^#^	Add ^N5^	[28]
Quercetin + resveratrol, 100 µg/mL	TRAP	64.9 ^#^	Ant ^N10^	[54]
Caffeic acid + α-tocopherol:	β-Carotene bleaching			[53]
0.2:1		96	Add ^N10^	
0.5:1		96	Add ^N10^	
1:1		97	Add ^N10^	
2:1		95	Add ^N10^	
Quercetin + α-tocopherol:	β-Carotene bleaching			[53]
0.5:1		163	Syn ^N10^	
1:1		109	Add ^N10^	
2:1		91	Add ^N10^	
5:1		108	Add ^N10^	
Up to 5 µM α-Tocopherol+ equimolar caffeic acid	Inhibition of AAPH-induced oxidation of linoleic acid, lag time	81	Ant ^N10^	[55]
Up to 11 µM Quercetin + equimolar rosmarinic acid	Inhibition of AAPH-induced oxidation of linoleic acid, lag time	113	Syn ^N10^	[55]
4.5 µM Astaxanthin + 4.5 µM β-carotene	Inhibition of AMVN-induced oxidation of linoleic acid, lag time	146.8 ^#^	Syn ^N10^	[56]
4.5 µM Astaxanthin + 4.5 µM lycopene	Inhibition of AMVN-induced oxidation of linoleic acid, lag time	160.5 ^#^	Syn^N10^	[56]
4.5 µM Astaxanthin + 4.5 µM zeaxanthin	Inhibition of AMVN-induced oxidation of linoleic acid, lag time	93.8 ^#^	Add ^N10^	[56]
1 mM Curcumin + 1 mM (−)epicatechin	Inhibition of sunflower oil triacylglycerol autoxidation at 80 °C	102.4 ^#^	Add ^T^	[57]
1 µM Resveratrol + 1 µM PMHC, pH 4.0	Inhibition of AAPH-induced DMPC oxidation, lag time	114.1 ^#^	Syn ^N10^	[58]
1 µM Resveratrol + 1 µM PMHC, pH 6.0	Inhibition of AAPH-induced oxidation of methyl linoleate in DMPC liposomes, lag time	116.2 ^#^	Syn ^N10^	[58]
1 µM Resveratrol + 1 µM PMHC, pH 7.0	Inhibition of AAPH-induced oxidation of methyl linoleate in DMPC liposomes, lag time	174.4 ^#^	Syn ^N10^	[58]
1 µM Resveratrol + 1 µM PMHC, pH 8.0	Inhibition of AAPH-induced oxidation of methyl linoleate in DMPC liposomes, lag time	91.0 ^#^	Add ^N10^	[58]
7.5 µM α-Tocopherol + 10 µM (−)epigallocatechin gallate + 10 µM ascorbic acid	Oxidation of linoleic acid in SDS micelles, lag time	200.0 ^#^	Syn ^N10^	[59]
7.5 µM α-Tocopherol + 10 µM (−)epicatechin gallate + 10 µM ascorbic acid	Oxidation of linoleic acid in SDS micelles, lag time	143.5 ^#^	Syn ^N10^	[59]
7.5 µM α-Tocopherol + 10 µM (−)epigallocatechin + 10 µM ascorbic acid	Oxidation of linoleic acid in SDS micelles, lag time	147.1 ^#^	Syn ^N10^	[59]
77 µM Ascorbic acid + 77 µM chlorogenic acid + 77 µM cysteine	Superoxide scavenging EPR, DMPO spin trap	104.5 ^#^	Add ^N10^	[60]
50 µM Ascorbic acid + 50 µM chlorogenic acid + 50 µM cysteine	*t*-Butyl peroxide scavenging EPR, DMPO spin trap	265.0 ^#^	Syn ^N10^	[60]
476 µM Ascorbic acid + 476 µM chlorogenic acid	Photoelectrochemical sensor, amperometric	235	Syn ^N10^	[61]
476 µM Ascorbic acid + 476 µM gallic acid	Photoelectrochemical sensor, amperometric	195	Syn ^N10^	[61]
476 µM Ascorbic acid + 476 µM proanthocyanidins	Photoelectrochemical sensor, amperometric	186	Syn ^N10^	[61]
8 µM * (Lycopene + quercetin), Molar ratio 1:10	Cellular antioxidant activity, HUVECs	120.2 ^#^	Syn ^A^	[62]
8 µM * (Lycopene + quercetin), Molar ratio 1:5	Cellular antioxidant activity, HUVECs	147.9 ^#^	Syn ^A^	[62]
8 µM * (Lycopene + quercetin), Molar ratio 1:1	Cellular antioxidant activity, HUVECs	160.9 ^#^	Syn ^A^	[62]
8 µM * (Lycopene + quercetin), Molar ratio 5:1	Cellular antioxidant activity, HUVECs	201.9 ^#^	Syn ^A^	[62]
8 µM * (Lycopene + quercetin), Molar ratio 10:1	Cellular antioxidant activity, HUVECs	164.7 ^#^	Syn ^A^	[62]
8 µM * (Lycopene + quercetin), Molar ratio 1:10	Cellular antioxidant activity, L-02 cells	112.4 ^#^	Syn ^A^	[62]
8 µM * (Lycopene + quercetin), Molar ratio 1:5	Cellular antioxidant activity, L-02 cells	117.7 ^#^	Syn ^A^	[62]
8 µM * (Lycopene + quercetin), Molar ratio 1:1	Cellular antioxidant activity, L-02 cells	100.7 ^#^	Add ^A^	[62]
8 µM * (Lycopene + quercetin), Molar ratio 5:1	Cellular antioxidant activity, L-02 cells	79.7 ^#^	Ant ^A^	[62]
8 µM * (Lycopene + quercetin), Molar ratio 10:1	Cellular antioxidant activity, L-02 cells	93.2 ^#^	Add ^A^	[62]

The concentrations indicated are final concentrations in the reaction mixture. ^#^ Interaction parameter calculated based on primary data presented in a publication. AAPH, 2,2′-azobis(2-amidinopropane) dihydrochloride; AMVN, 2,2′-azobis(2,4-dimethylvaleronitrile); DMPC, dimirystoylphosphatidylcholine; DMPO, 5,5-dimethyl-1-pyrroline *N*-oxide; GSH, glutathione; HUVECs, human umbilical vein endothelial cells; PMHC, 2,2,5,7,8-ppentamethyl-6-hydrochromanol. Add, additivity; Ant, antagonism; Syn, Synergy. ^A^, statistical evaluation, one-way ANOVA; ^T^; statistical evaluation, Student’s “*t*” test; ^N10^, no statistical evaluation; the criterion of 10% assumed; ^N5^, no statistical evaluation; the criterion of 5% assumed. * Concentration used for cell treatment; intracellular concentration not determined.

**Table 3:** Exemplary qualitative results of interactions between antioxidants in vitro.

System	Assay	Effects	Reference
Taxifolin + GSH, quercetin + GSH, rutin + GSH, morin + GSH	ABTS^•^ decolorization	Antagonism or additivity (“classic” analysis) Synergy or additivity Webb’s simulation) ^T^	[20]
Chlorogenic acid + gallic acid	ABTS^•^ decolorization	Synergy (111–135% of additive activity) ^A^	[63]
Caffeic acid + gallic acid	ABTS^•^ decolorization	Synergy (113–142% of additive activity) ^A^	[63]
S-Allyl-L-cysteine + catechin	ABTS^•^ decolorization	Antagonism (CI > 1) ^D^	[64]
S-Allyl-L-cysteine + quercetin		Antagonism (CI > 1) ^D^	
S-Allyl-L-cysteine + caffeic acid		Antagonism (CI > 1) ^D^	
S-Allyl-L-cysteine + ferulic acid		Antagonism (CI > 1) ^D^	
S-Allyl-L-cysteine + sinapic acid		Antagonism (CI > 1) ^D^	
S-Allyl-L-cysteine + 3,4-dihydroxybenzoic acid		Antagonism (CI > 1) ^D^	
Curcumin + quercetin	ABTS^•^ decolorization, DPPH^•^ decolorization	Antagonism, decreasing with increasing the quercetin/curcumin ratio from 1:4 to 4:1 ^A^	[65]
Rutin + resveratrol 2:1, 3.3–16.7 µM	DPPH^•^ decolorization	Antagonistic, % decolorization for mixture lower than that for one of the components ^A^	[66]
Ascorbic acid + trehalose, catechin + trehalose, gallic acid + trehalose, quercetin + trehalose	DPPH^•^ decolorization	Antagonistic interaction, CI increasing with rising concentration of antioxidants. The role of trehalose unclear (it does not reduce DPPH^•^) ^T^	[67]
α-Tocopherol + γ-oryzanol,	DPPH^•^ decolorization	Synergy (20 + 200, 40 + 400, 60 + 600, 80 + 800, 1000 + 10,000 mg/kg) ^A^	[68]
γ-Oryzanol + phytosterol in refined coconut oil	DPPH^•^ decolorization	Additivity (20 + 200 mg/kg) Synergy (40 + 400, 60 + 600, 80 + 800, 1000 + 10,000 mg/kg) ^A^	[68]
α-Tocopherol + phytosterol in refined coconut oil	DPPH^•^ decolorization	Synergy (20 + 200, 40 + 400, 60 + 600, 80 + 800, 1000 + 10,000 mg/kg) ^A^	[68]
α-Tocopherol + γ-oryzanol in purified rice bran oil	DPPH^•^ decolorization	Antagonism (50 + 2000, 100 + 4000, 200 + 8000, 400 + 10,000, 500 + 12,000 mg/kg) ^A^	[69]
α-Tocopherol + phytosterol in purified rice bran oil	DPPH^•^ decolorization	Antagonism (50 + 2000, 100 + 4000, 200 + 8000, 400 + 10,000, 500 + 12,000 mg/kg) ^A^	[69]
γ-Oryzanol + phytosterol in purified rice bran oil	DPPH^•^ decolorization	Synergy (2000 + 2000, 4000 + 4000, 8000 + 8000, 10,000 + 10,000 and 12,000 + 12,000 mg/kg) ^A^	[69]
Quercetin + esculetin, caffeoyl tartaric acid + gentisic acid, caffeoyl tartaric acid + esculetin, esculetin + gentisic acid, quercetin 3-O-arabinoside + gentisic acid, quercetin + gentisic acid, quercetin 3-O-arabinoside	DPPH^•^ decolorization	Synergy (CI < 1) ^A^	[70]
Quercetin + caffeoyl tartaric acid, quercetin 3-O-arabinoside + caffeoyl tartaric acid, rhamnetin + cyanidin 3-O-galactoside, quercetin + cyanidin 3-O-galactoside, quercetin 3-O-arabinoside + cyanidin 3-O-galactoside, quercetin 3-O-arabinoside + rhamnetin, cyanidin 3-O-galactoside + caffeoyl tartaric acid, cyanidin 3-O-galactoside + esculetin	DPPH^•^ decolorization	Antagonism (CI > 1) ^A^	[70]
Flavonoids	DPPH^•^ decolorization	Antagonistic, additive or synergistc effects (from 11.9% to 121.7%)	[71]
S-Allyl-L-cysteine + catechin	DPPH^•^ decolorization	Synergy (CI < 1) ^TK^	[64]
S-Allyl-L-cysteine + quercetin		Synergy (CI < 1) ^TK^	
S-Allyl-L-cysteine + caffeic acid		Synergy (CI < 1) ^TK^	
S-Allyl-L-cysteine + ferulic acid		Synergy (CI < 1) ^TK^	
S-Allyl-L-cysteine + sinapic acid		Synergy (CI < 1) ^TK^	
S-Allyl-L-cysteine + 3,4-dihydroxybenzoic acid		Synergy (CI < 1) ^TK^	
Flavonoids	FRAP	Antagonistic, additive or synergistic effects (from 91.9.1% to 122.4%)	[71]
S-Allyl-L-cysteine + catechin	Ferricyanide reduction	Synergy (CI < 1) ^D^	[64]
S-Allyl-L-cysteine + quercetin		Synergy (CI < 1) ^D^	
S-Allyl-L-cysteine + caffeic acid		Synergy (CI < 1) ^D^	
S-Allyl-L-cysteine + ferulic acid		Antagonism (CI > 1) ^D^	
S-Allyl-L-cysteine + sinapic acid		Synergy (CI < 1) ^D^	
S-Allyl-L-cysteine + 3,4-dihydroxybenzoic acid		Additivity (CI ≈ 1) ^D^	
Resveratrol + caffeic acid, equimolar	Ferricyanide reduction	Additivity (CI ≈ 1) ^N^	[72]
Quercetin + resveratrol, equimolar Quercetin + caffeic acid, equimolar	Nonsite-specific hydroxyl radical-mediated 2-deoxy-D-ribose degradation, ferric ion reducing power	Synergy (CI < 1) ^N^	[72]
Resveratrol + caffeic acid, equimolar	Nonsite-specific hydroxyl radical-mediated 2-deoxy-D-ribose degradation, FRAP, NO^•^ scavenging	Synergy(CI < 1) ^N^	[72]
Quercetin + resveratrol + caffeic acid, equimolar	Nonsite-specific hydroxyl radical-mediated 2-deoxy-D-ribose degradation, ferric reducing power, FRAP, NO^•^ scavenging	Synergy(CI < 1) ^N^	[72]
Resveratrol + caffeic acid, equimolar	Site-specific hydroxyl radical-mediated 2-deoxy-D-ribose degradation, DPPH^•^ decolorization, ABTS^•^ decolorization	Antagonism (CI > 1) ^N^	[72]
Quercetin + resveratrol + caffeic acid, equimolar	Site-specific hydroxyl radical-mediated 2-deoxy-D-ribose degradation, DPPH^•^ decolorization, ABTS^•^ decolorization	Antagonism (CI > 1) ^N^	[72]
Quercetin + α-tocopherol	Oxidation of methyl linoleate, reaction half-time	Synergy or additivity ^A2^	[73]
Quercetin + astaxanthin	Oxidation of methyl linoleate, reaction half-time	Additivity ^A2^	[43]
Quercetin + rutin	Oxidation of methyl linoleate, reaction half-time	Synergy ^A2^	[73]
γ-Terpinene + PMHC or CAPE	Oxidation of styrene, lag time	Prolongation of the induction time by γ-terpinene, which did not show any induction time itself ^N^	[74]
Quercetin + rutin	Peroxidation of sunflower oil, lag time	Additivity ^A2^	[73]
RA + CHA, RA + EC, RA + AA, RA + GA, RA + PC, EC + PC, CHA + PC, CHA + EC, EC + AA, CHA + GA, GA + PC, PC + GA + AA + EC + CHA + RA PC + AA + EC + CHA + RA PC + GA + EC + CHA + RA PC + GA + AA + CHA + RA GA + AA + EC + CHA + RA PC + GA + AA + EC + RA PC + GA + AA + EC + CHA	Photochemical electrode, amperometric	Synergy or antagonism ^N^	[75]
α-Tocopherol + γ-oryzanol, in refined coconut oil	Lag time (Rancimat)	Synergy (20 + 200, 40 + 400, 60 + 600, 80 + 800, 1000 + 10,000 mg/kg) ^A^	[68]
α-Tocopherol + phytosterol in refined coconut oil	Lag time (Rancimat)	Antagonism (20 + 200 mg/kg) Synergy (40 + 400, 60 + 600, 80 + 800, 1000 + 10,000 mg/kg) ^A^	[68]
γ-Oryzanol + phytosterol in refined coconut oil	Lag time (Rancimat)	Antagonism (20 + 200 mg/kg) Synergy (40 + 400, 60 + 600, 80 + 800, 1000 + 10,000 mg/kg) ^A^	[68]
α-Tocopherol + γ-oryzanol in purified rice bran oil	Lag time (Rancimat)	Antagonism (50 + 2000, 100 + 4000, 200 + 8000, 400 + 10,000, 500 + 12,000 mg/kg) ^A^	[69]
α-Tocopherol + phytosterol in purified rice bran oil	Lag time (Rancimat)	Antagonism (50 + 2000, 100 + 4000, 400 + 10,000, 500 + 12,000 mg/kg) or additivity (200 + 8000 mg/kg) ^A^	[69]
γ-Oryzanol + phytosterol in purified rice bran oil	Lag time (Rancimat)	Synergy (2000 + 2000, 8000 + 8000, 10,000 + 10,000 and 12,000 + 12,000 mg/kg) Antagonism (4000 + 4000 mg/kg) ^A^	[69]
Caffeoyl tartaric acid + gentisic acid, quercetin + quercetin 3-O-arabinoside, quercetin + caffeoyl tartaric acid;	Lard oxidation, lag time (Rancimat)	Synergy (CI < 1) ^A^	[70]
α-Tocopherol + rosmarinic acid	AAPH-induced oxidation of linoleic acid, inhibition of peroxide formation	Synergy (CI < 1) ^TK^	[76]
α-Tocopherol + rosmarinic acid	AAPH-induced oxidation of linoleic acid, inhibition of hexanal formation	Synergy (CI < 1) ^TK^	[76]
α-Tocopherol + caffeic acid		Synergy (CI < 1) ^TK^	
α-Tocopherol + luteolin		Synergy (CI < 1) ^TK^	
α-Tocopherol + rosmarinic acid	AAPH-induced oxidation of linoleic acid, inhibition of *(E)*-2-heptanal formation	Synergy (CI < 1) ^TK^	[76]
α-Tocopherol + caffeic acid		Additivity (CI ≈ 1) ^TK^	
α-Tocopherol + luteolin		Synergy (CI < 1) ^TK^	
α-Tocopherol + rosmarinic acid	AAPH-induced oxidation of linoleic acid, inhibition of *(E)*-2-nonenal formation	Synergy (CI < 1) ^TK^	[76]
α-Tocopherol + caffeic acid		Synergy (CI < 1) ^TK^	
α-Tocopherol + luteolin		Synergy (CI < 1) ^TK^	
α-Tocopherol + rosmarinic acid	AAPH-induced oxidation of linoleic acid, inhibition of *(E,E)*-2,4-decadienal formation	Antagonism (CI > 1) ^TK^	[76]
α-Tocopherol + caffeic acid		Additivity (CI ≈ 1) ^TK^	
α-Tocopherol + luteolin		Synergy (CI < 1) ^TK^	
EGCG + kaempferol	Cellular antioxidant activity		[77]
6:1.5		Synergy (CI < 1) ^AD^	
7:1.5		Antagonism (CI > 1) ^AD^	

Interaction parameter < 100%, antagonism; interaction parameter > 100%, synergy. AA, ascorbic acid; CAPE, caffeic acid phenethyl ester; CHA, catechinic acid; EC, epicatechin; GA, gallic acid; GSH, glutathione; PC, proanthocyanidins; PMHC, 2,2,5,7,8-pentamethyl-6-chromanol; RA, resveratrol. Green color indicates synergistic interactions, red color indicates antagonistic interactions; ^N^, no statistics; ^A^, ANOVA; ^A2^; two-way ANOVA; ^D^, Duncan’s multiple range tests; ^TK^, Tukey–Kramer test.

**Table 4:** Exemplary quantitative results of interactions between food products in vitro.

System	Assay	Interaction Parameter = 100% × [(Effect Measured)/(Effect Predicted as the Arithmetic Sum of Individual Antioxidant Activities)]; Type of Interaction		Reference
32% fresh spirulina/68% apple juice	ABTS^•^ decolorization	60	Ant ^N10^	[136]
32% fresh spirulina, 43% Japanese quince juice, 25% sugar	ABTS^•^ decolorization	97	Add ^N10^	[136]
32% fresh spirulina, 43% Japanese quince juice, 25% sugar	ABTS^•^ decolorization	97	Add ^N10^	[136]
Jute + blackjack 3:7	ABTS^•^ decolorization	142.9	Syn ^N10^	[145]
Jute + blackjack 5:5	ABTS^•^ decolorization	104.0	Add ^N10^	[145]
Jute + blackjack 7:3	ABTS^•^ decolorization	100.9	Add ^N10^	[145]
Breakfast cereals + strawberry	Q/ABTS^•^ decolorization	158.9	Syn ^T^	[144]
		BF 92.4	Add ^T^	
Breakfast cereals + blueberry	Q/ABTS^•^ decolorization	128.7	Syn ^T^	[144]
Breakfast cereals + black grape	Q/ABTS^•^ decolorization	160.5	Syn ^T^	[144]
Breakfast cereals + hazelnut	Q/ABTS^•^ decolorization	153.6	Syn ^T^	[144]
		BF 40.7	Ant ^T^	
Breakfast cereals + chia seed	Q/ABTS^•^ decolorization	109.4	Add ^T^	[144]
Breakfast cereals + flaxseed	Q/ABTS^•^ decolorization	101.9	Add ^T^	[144]
		BF 88.1	Ant ^T^	
Breakfast cereals + yoghurt	Q/ABTS^•^ decolorization	118.1	Syn ^T^	[144]
Breakfast cereals + milk	Q/ABTS^•^ decolorization	35.3	Ant ^T^	[144]
		BF 118.8	Syn ^T^	
White wheat bread + green tea extract	Q/ABTS^•^ decolorization	139.1	Syn ^T^	[144]
		BF 38.9	Ant ^T^	
White wheat bread + black tea extract	Q/ABTS^•^ decolorization	173.1	Syn ^T^	[144]
		BF 123.7	Syn ^T^	
White wheat bread + flaxseed	Q/ABTS^•^ decolorization	81.7	Ant ^T^	[144]
		BF 72.4	Ant ^T^	
White wheat bread + chia seed	Q/ABTS^•^ decolorization	67.9	Ant ^T^	[144]
White wheat bread + sesame	Q/ABTS^•^ decolorization	72.5	Ant ^T^	[144]
White wheat bread + cheese	Q/ABTS^•^ decolorization	91.0	Add ^T^	[144]
White wheat bread + milk	Q/ABTS^•^ decolorization	73.5	Ant ^T^	[144]
Milk + strawberry	Q/ABTS^•^ decolorization	73.6	Ant ^T^	[144]
		BF 135.2	Syn ^T^	
Milk + blueberry	Q/ABTS^•^ decolorization	70.6	Ant ^T^	[144]
Milk + black grape	Q/ABTS^•^ decolorization	81.5	Ant ^T^	[144]
Yoghurt + strawberry	Q/ABTS^•^ decolorization	118.3	Syn ^T^	[144]
Yoghurt + blueberry	Q/ABTS^•^ decolorization	156.5	Syn ^T^	[144]
Yoghurt + black grape	Q/ABTS^•^ decolorization	160.9	Syn ^T^	[144]
Milk + chia seed	Q/ABTS^•^ decolorization	43.7	Ant ^T^	[144]
Milk + flaxseed	Q/ABTS^•^ decolorization	54.7	Ant ^T^	[144]
		BF 45.3	Ant ^T^	
Yoghurt + flaxseed	Q/ABTS^•^ decolorization	166.4	Syn ^T^	[144]
Yoghurt + chia seed	Q/ABTS^•^ decolorization	100.2	Add ^T^	[144]
Milk + green tea extract	Q/ABTS^•^ decolorization	89.0	Ant ^T^	[144]
		BF 40.8	Ant ^T^	
Milk + black tea extract	Q/ABTS^•^ decolorization	234.4	Syn ^T^	[144]
		BF 65.2	Ant ^T^	
Milk + espresso	Q/ABTS^•^ decolorization	162.6	Syn ^T^	[144]
		BF 87.7	Ant ^T^	
Jambon + white wheat bread	Q/ABTS^•^ decolorization	83.8	Ant ^T^	[144]
		BF 112.4	Syn ^T^	
Jambon + lettuce	Q/ABTS^•^ decolorization	77.6	Ant ^T^	[144]
Jambon + tomato	Q/ABTS^•^ decolorization	76.4	Ant ^T^	[144]
Jambon + cheese	Q/ABTS^•^ decolorization	146.2	Syn ^T^	[144]
		BF 99.9	Add ^T^	
Lettuce + tomato	Q/ABTS^•^ decolorization	135.6	Syn ^T^	[144]
Lettuce + cabbage	Q/ABTS^•^ decolorization	113.0	Syn ^T^	[144]
Tomato + white wheat bread	Q/ABTS^•^ decolorization	119.2	Syn ^T^	[144]
Tomato + cheese	Q/ABTS^•^ decolorization	119.6	Syn ^T^	[144]
Wine + tomato	Q/ABTS^•^ decolorization	117.3	Syn ^T^	[144]
Wine + white wheat bread	Q/ABTS^•^ decolorization	114.3	Syn ^T^	[144]
		BF 75.2	Ant ^T^	
Wine + cheese	Q/ABTS^•^ decolorization	122.2	Syn ^T^	[144]
		BF 94.9	Add ^T^	
Wine + jambon	Q/ABTS^•^ decolorization	142.1	Syn ^T^	[144]
		BF 124.9	Add ^T^	
Jute + blackjack 3:7	DPPH^•^ decolorization	113.0	Syn ^N10^	[145]
Jute + blackjack 5:5	DPPH^•^ decolorization	109.2	Add ^N10^	[145]
Jute + blackjack 7:3	DPPH^•^ decolorization	101.9	Add ^N10^	[145]
Jute + blackjack 3:7	FRAP	89.4	Ant ^N10^	[145]
Jute + blackjack 5:5	FRAP	83.9	Ant ^N10^	[145]
Jute + blackjack 7:3	FRAP	82.0	Ant ^N10^	[145]
Ethanolic extracts of leaves of *Mangifera indica* + extracts of the aerial parts of *Euphorbia hirta*	FRAP			[139]
1:3		94.2	Add ^N10^	
1:1		78.8	Ant ^N10^	
Green tea extract + honey	FRAP	121.7	Syn ^A^	[135]
Green tea extract + honey + lemon extract	FRAP	180.0	Syn ^A^	[135]
32% fresh spirulina/68% apple juice	FRAP	102	Add ^N10^	[136]
32% fresh spirulina, 43% Japanese quince juice, 25% sugar	FRAP	131	Syn ^N10^	[136]
32% fresh spirulina/68% apple juice	Total phenolics	77	Ant ^N10^	[136]
32% fresh spirulina, 43% Japanese quince juice, 25% sugar	Total phenolics	74	Ant ^N10^	[136]

Interaction parameter < 100%, antagonism; interaction parameter > 100%, synergy. BF, bioaccessible fraction; Q, quencher approach. Add, additivity; Ant, antagonism; Syn, synergy; ^N5^, criterion of 5% difference from 100%; ^N10^, criterion of 10% difference from 100%. ^A^, ANOVA; ^T^, Student’s “*t*” test.

**Table 5:** Exemplary qualitative results of interactions between food products in vitro.

System	Assay	Effects	Reference
Curcumin + epicatechin fraction of green tea	ABTS^•^ decolorization	Synergy (CI 0.80–0.91) ^A^	[28]
TEMPOL + red cabbage extract	ABTS^•^ decolorization	Antagonism ^T^	[78]
Trolox + red cabbage extract	ABTS^•^ decolorization	Additivity ^T^	[78]
*Lavandula stoechas* essential oil + *Mentha pulegium* essential oil, 1:2, 1:1.2:1	ABTS^•^ decolorization	Antagonism (1:2, 1:1) ^A^ Synergy (2:1)	[141]
*Laurus nobilis* essential oil + *Lavandula stoechas* essential oil, 1:2, 1:1.2:1	ABTS^•^ decolorization	Synergy (CI 0.15–0.54) ^A^	[141]
*Laurus nobilis* essential oil + *Mentha pulegium* essential oil, 1:2, 1:1.2:1	ABTS^•^ decolorization	Synergy (CI 0.16–0.28) ^A^	[141]
*Lavandula stoechas* essential oil + *Laurus nobilis* essential oil + *Mentha pulegium* essential oil, 1:1:1, 2:1:1, 1:2:1, 1:1:2	ABTS^•^ decolorization	Synergy (CI 0.25–0.97) ^A^	[141]
*Lavandula stoechas* essential oil + *Mentha pulegium* essential oil, 1:2, 1:1.2:1	DPPH^•^ decolorization	Antagonism (CI 1.65–7.04) ^A^	[141]
*Laurus nobilis* essential oil + *Lavandula stoechas* essential oil, 1:2, 1:1.2:1	DPPH^•^ decolorization	Synergy (CI 0.21–0.40) ^A^	[141]
*Laurus nobilis* essential oil + *Mentha pulegium* essential oil, 1:2, 1:1.2:1	DPPH^•^ decolorization	Synergy (CI 0.24–0.40) ^A^	[141]
*Lavandula stoechas* essential oil + *Laurus nobilis* essential oil + *Mentha pulegium* essential oil, 1:1:1, 2:1:1, 1:2:1, 1:1:2	DPPH^•^ decolorization	Synergy (CI 0.23–0.75) ^A^	[141]
Glutathione + red cabbage extract	FRAP	Synergy ^T^	[78]
Ascorbic acid + red cabbage extract	FRAP	Additivity ^T^	[78]
Trolox + red cabbage extract	FRAP	Additivity ^T^	[78]
*Lavandula stoechas* essential oil + *Mentha pulegium* essential oil, 1:2, 1:1.2:1	CUPRAC	Antagonism (1:2, 1:1) ^A^ Synergy (2:1) ^A^	[141]
*Laurus nobilis* essential oil + *Lavandula stoechas* essential oil, 1:2, 1:1.2:1	CUPRAC	Synergy (CI 0.03–0.19) ^A^	[141]
*Laurus nobilis* essential oil + *Mentha pulegium* essential oil, 1:2, 1:1.2:1	CUPRAC	Synergy (CI 0.03–0.27) ^A^	[141]
*Lavandula stoechas* essential oil + *Laurus nobilis* essential oil + *Mentha pulegium* essential oil, 1:1:1, 2:1:1, 1:2:1, 1:1:2	CUPRAC	Antagonism (1:1:1) ^A^ Synergy (2:1:1, 1:2:1, 1:1:2) ^A^	[141]
Trolox + red cabbage extract	ORAC	Synergy ^T^	[79]
TEMPOL + red cabbage extract	ORAC	Antagonism ^T^	[79]

^A^, ANOVA; ^T^, Student’s “*t*” test.

## Data Availability

No new data were created or analyzed in this study. Data sharing is not applicable to this article.

## Abbreviations

The following abbreviations are used in this manuscript:

AAPH	2,2′-Azobis(2-amidinopropane) dihydrochloride
ABTS	2,2′-Azino-bis(3-ethylbenzothiazoline-6-sulfonic acid)
AMVN	2,2′-Azobis(2,4-dimethylvaleronitrile)
BHT	Butylated hydroxytoluene
CAPE	Caffeic acid phenethyl ester
CI	Combination Index
CTAB	Cetrimonium bromide
DMPC	Dimyristoylphosphatidylcholine
DMPO	5,5-Dimethyl-1-pyrroline N-oxide
DPPH	2,2-Diphenyl-1-picrylhydrazyl
ECG	Epicatechin gallate
EGCG	Epigallocatechin gallate
EPR	Electron paramagnetic resonance
FRAP	Ferric Reducing Antioxidant Power
GSH	Glutathione
HUVECs	Human umbilical vein endothelial cells
ORAC	Oxygen Radical Absorbance Capacity
PMHC	2,2,5,7,8-Pentamethyl-6-hydrochromanol
SDS	Sodium Dodecyl Sulfate
TBHQ	*tert*–Butyl hydroxyquinone

## References

[1] Harman D. (1956). Aging: A theory based on free radical and radiation chemistry. J. Gerontol..

[2] Polidori M.C., Mecocci P. (2022). Modeling the dynamics of energy imbalance: The free radical theory of aging and frailty revisited. Free Radic. Biol. Med..

[3] Singh V., Chandra N., Gupta V., Gautam P., Rath S., Sethi A.K., Chaurasia R.N., Mishra V.N., Rai S.N. (2025). Oxidative stress and aging. Neurobiology of Aging.

[4] Barbouti A., Goulas V. (2021). Dietary antioxidants in the Mediterranean diet. Antioxidants.

[5] Gantenbein K.V., Kanaka-Gantenbein C. (2021). Mediterranean diet as an antioxidant: The impact on metabolic health and overall wellbeing. Nutrients.

[6] Kiani A.K., Medori M.C., Bonetti G., Aquilanti B., Velluti V., Matera G., Iaconelli A., Stuppia L., Connelly S.T., Herbst K.L. (2022). Modern vision of the Mediterranean diet. J. Prev. Med. Hyg..

[7] Teleanu D.M., Niculescu A.G., Lungu I.I., Radu C.I., Vladâcenco O., Roza E., Costăchescu B., Grumezescu A.M., Teleanu R.I. (2022). An overview of oxidative stress, neuroinflammation, and neurodegenerative diseases. Int. J. Mol. Sci..

[8] Jomova K., Raptova R., Alomar S.Y., Alwasel S.H., Nepovimova E., Kuca K., Valko M. (2023). Reactive oxygen species, toxicity, oxidative stress, and antioxidants: Chronic diseases and aging. Arch. Toxicol..

[9] Reddy V.P. (2023). Oxidative stress in health and disease. Biomedicines.

[10] Konfo T.R.C., Djouhou F.M.C., Koudoro Y.A., Dahouenon-Ahoussi E., Avlessi F., Sohounhloue C.K.D., Simal-Gandara J. (2023). Essential oils as natural antioxidants for the control of food preservation. Food Chem. Adv..

[11] Eze C.N., Aduba C.C., Ezema B.O., Ayoka T.O., Nnadi C.O., Onyeaka H. (2025). The role of antioxidants in food safety and preservation: Mechanisms, applications, and challenges. Cogent Food Agric..

[12] Parveen B., Rajinikanth V., Narayanan M. (2025). Natural plant antioxidants for food preservation and emerging trends in nutraceutical applications. Discov. Appl. Sci..

[13] Hannan P.A., Khan J.A., Ullah I., Ullah S. (2016). Synergistic combinatorial antihyperlipidemic study of selected natural antioxidants; modulatory effects on lipid profile and endogenous antioxidants. Lipids Health Dis..

[14] Chen X., Li H., Zhang B., Deng Z. (2022). The synergistic and antagonistic antioxidant interactions of dietary phytochemical combinations. Crit. Rev. Food Sci. Nutr..

[15] Townsend J.R., Kirby T.O., Sapp P.A., Gonzalez A.M., Marshall T.M., Esposito R. (2023). Nutrient synergy: Definition, evidence, and future directions. Front. Nutr..

[16] Hajimehdipoor H., Shahrestani R., Shekarchi M. (2014). Investigating the synergistic antioxidant effects of some flavonoid and phenolic compounds. Res. J. Pharmacogn..

[17] Bayram I., Decker E.A. (2023). Underlying mechanisms of synergistic antioxidant interactions during lipid oxidation. Trends Food Sci. Technol..

[18] Gao X., Xiao Y., Li W., Xu L., Yuan J. (2025). Synergistic effects of antioxidant blends: A comparative study on oxidative stability of lipids in feed matrices. Antioxidants.

[19] Parcheta M., Świsłocka R., Orzechowska S., Akimowicz M., Choińska R., Lewandowski W. (2021). Recent developments in effective antioxidants: The structure and antioxidant properties. Materials.

[20] Ilyasov I., Beloborodov V., Antonov D., Dubrovskaya A., Terekhov R., Zhevlakova A., Saydasheva A., Evteev V., Selivanova I. (2020). Flavonoids with glutathione antioxidant synergy: Influence of free radicals inflow. Antioxidants.

[21] Zhao L., Wientjes M.G., Au J.L. (2004). Evaluation of combination chemotherapy: Integration of nonlinear regression, curve shift, isobologram, and combination index analyses. Clin. Cancer Res..

[22] Chou T.C. (2006). Theoretical basis, experimental design, and computerized simulation of synergism and antagonism in drug combination studies. Pharmacol. Rev..

[23] Roell K.R., Reif D.M., Motsinger-Reif A.A. (2017). An introduction to terminology and methodology of chemical synergy—Perspectives from across disciplines. Front. Pharmacol..

[24] Chou T.C. (2018). The combination index (CI < 1) as the definition of synergism and of synergy claims. Synergy.

[25] Greco W.R., Bravo G., Parsons J.C. (1995). The search for synergy: A critical review from a response surface perspective. Pharmacol. Rev..

[26] Webb J.L. (1963). Effect of more than one inhibitor. Enzyme and Metabolic Inhibitors.

[27] Pereira R.B., Sousa C., Costa A., Andrade P.B., Valentão P. (2013). Glutathione and the antioxidant potential of binary mixtures with flavonoids: Synergisms and antagonisms. Molecules.

[28] Skroza D., Šimat V., Vrdoljak L., Jolić N., Skelin A., Čagalj M., Frieta R., Generalić Mekinić I. (2022). Investigation of antioxidant synergisms and antagonisms among phenolic acids in the model matrices using FRAP and ORAC methods. Antioxidants.

[29] Twarog N.R., Stewart E., Hammill C.V., Shelat A.A. (2016). BRAID: A unifying paradigm for the analysis of combined drug action. Sci. Rep..

[30] Lee J.J., Kong M. (2009). Confidence intervals of interaction index for assessing multiple drug interaction. Stat. Biopharm. Res..

[31] Re R., Pellegrini N., Proteggente A., Pannala A., Yang M., Rice-Evans C. (1999). Antioxidant activity applying an improved ABTS radical cation decolorization assay. Free Radic. Biol. Med..

[32] Kut K., Cieniek B., Stefaniuk I., Bartosz G., Sadowska-Bartosz I. (2022). A modification of the ABTS^•^ decolorization method and an insight into its mechanism. Processes.

[33] Erel O. (2004). A novel automated direct measurement method for total antioxidant capacity using a new generation, more stable ABTS radical cation. Clin. Biochem..

[34] Kedare S.B., Singh R.P. (2011). Genesis and development of DPPH method of antioxidant assay. J. Food Sci. Technol..

[35] Gulcin İ., Alwasel S.H. (2023). DPPH radical scavenging assay. Processes.

[36] Yeo J., Shahidi F. (2019). Critical re-evaluation of DPPH assay: Presence of pigments affects the results. J. Agric. Food Chem..

[37] Benzie I.F., Strain J.J. (1996). The ferric reducing ability of plasma (FRAP) as a measure of “antioxidant power”: The FRAP assay. Anal. Biochem..

[38] Benzie I.F., Devaki M., Apak R., Capanoglu E., Shahidi F. (2018). The ferric reducing/antioxidant power (FRAP) assay for non-enzymatic antioxidant capacity: Concepts, procedures, limitations and applications. Measurement of Antioxidant Activity & Capacity: Recent Trends and Applications.

[39] Ou B., Hampsch-Woodill M., Prior R.L. (2001). Development and validation of an improved oxygen radical absorbance capacity assay using fluorescein as the fluorescent probe. J. Agric. Food Chem..

[40] Prior R.L. (2015). Oxygen radical absorbance capacity (ORAC): New horizons in relating dietary antioxidants/bioactives and health benefits. J. Funct. Foods.

[41] Sadowska-Bartosz I., Bartosz G. (2026). ORAC: The method of choice for determining antioxidant capacity of food products?. Int. J. Mol. Sci..

[42] Marco G.J. (1968). A rapid method for evaluation of antioxidants. J. Am. Oil Chem. Soc..

[43] Prieto M.A., Rodríguez-Amado I., Vázquez J.A., Murado M.A. (2012). β-Carotene assay revisited. Application to characterize and quantify antioxidant and prooxidant activities in a microplate. J. Agric. Food Chem..

[44] Olszowy-Tomczyk M., Wianowska D. (2025). An updated overview on the use of the β-carotene bleaching method in assessing the antioxidant activity of compounds. Processes.

[45] Dufour C., Loonis M., Dangles O. (2007). Inhibition of the peroxidation of linoleic acid by the flavonoid quercetin within their complex with human serum albumin. Free Radic. Biol. Med..

[46] Villares A., Guillamón E., D’Arrigo M., Martínez J.A., García-Lafuente A., Ramos A. (2012). Kinetic study of the inhibition of linoleic acid oxidation in aqueous media by phenolic compounds. Food Biophys..

[47] Tiveron A.P., Melo P.S., Bergamaschi K.B., Vieira T.M., Regitano-d’Arce M.A., Alencar S.M. (2012). Antioxidant activity of Brazilian vegetables and its relation with phenolic composition. Int. J. Mol. Sci..

[48] Wolfe K.L., Liu R.H. (2007). Cellular antioxidant activity (CAA) assay for assessing antioxidants, foods, and dietary supplements. J. Agric. Food Chem..

[49] López-Alarcón C., Denicola A. (2013). Evaluating the antioxidant capacity of natural products: A review on chemical and cellular-based assays. Anal. Chim. Acta.

[50] Granato D. (2023). Cellular antioxidant activity measurement: An alternative to chemical antioxidant methods?. Food Saf. Health.

[51] Olszowy-Tomczyk M. (2020). Synergistic, antagonistic and additive antioxidant effects in the binary mixtures. Phytochem. Rev..

[52] Palafox-Carlos H., Gil-Chávez J., Sotelo-Mundo R.R., Namiesnik J., Gorinstein S., González-Aguilar G.A. (2012). Antioxidant interactions between major phenolic compounds found in ‘Ataulfo’ mango pulp: Chlorogenic, gallic, protocatechuic and vanillic acids. Molecules.

[53] Zhang Y., Wang X., Zeng Q., Deng Y., Xie P., Zhang C., Huang L. (2023). A new insight into synergistic effects between endogenous phenolic compounds additive and α-tocopherol for the stability of olive oil. Food Chem..

[54] Murugesan N., Damodaran C.P. (2023). Antioxidant activity of synergistic quercetin resveratrol. J. Mol. Chem..

[55] Peyrat-Maillard M.N., Cuvelier M.E., Berset C. (2003). Antioxidant activity of phenolic compounds in 2, 2′-azobis (2-amidinopropane) dihydrochloride (AAPH)-induced oxidation: Synergistic and antagonistic effects. J. Am. Oil Chem. Soc..

[56] Liang J., Tian Y.X., Yang F., Zhang J.P., Skibsted L.H. (2009). Antioxidant synergism between carotenoids in membranes. Astaxanthin as a radical transfer bridge. Food Chem..

[57] Slavova-Kazakova A., Janiak M.A., Sulewska K., Kancheva V.D., Karamać M. (2021). Synergistic, additive, and antagonistic antioxidant effects in the mixtures of curcumin with (−)-epicatechin and with a green tea fraction containing (−)-epicatechin. Food Chem..

[58] Konopko A., Litwinienko G. (2021). Unexpected role of pH and microenvironment on the antioxidant and synergistic activity of resveratrol in model micellar and liposomal systems. J. Org. Chem..

[59] Dai F., Chen W.F., Zhou B. (2008). Antioxidant synergism of green tea polyphenols with α-tocopherol and l-ascorbic acid in SDS micelles. Biochimie.

[60] Lo Scalzo R. (2021). EPR free radical scavenging activity on superoxide, hydroxyl and *tert*–butyl hydroperoxide radicals by common hydrophilic antioxidants: Effect of mixing and influence of glucose and citric acid. Eur. Food Res. Technol..

[61] Han F., Song Z., Xu J., Dai M., Luo S., Han D., Niu L., Wang Z. (2021). Oxidized titanium carbide MXene-enabled photoelectrochemical sensor for quantifying synergistic interaction of ascorbic acid based antioxidants system. Biosens. Bioelectron..

[62] Chen X., Deng Z., Zheng L., Zhang B., Luo T., Li H. (2021). Interaction between flavonoids and carotenoids on ameliorating oxidative stress and cellular uptake in different cells. Foods.

[63] Qi X., Liu H., Ren Y., Zhu Y., Wang Q., Zhang Y., Wu Y., Yuan L., Yan H., Liu M. (2023). Effects of combined binding of chlorogenic acid/caffeic acid and gallic acid to trypsin on their synergistic antioxidant activity, enzyme activity and stability. Food Chem. X.

[64] Dong C., Zhao G., Tao L., Qiu F., Wang S., Wang B., Liu J., Duan S. (2022). Antioxidant interactions between S-allyl-L-cysteine and polyphenols using interaction index and isobolographic analysis. Molecules.

[65] Jain S., Lenaghan S., Dia V., Zhong Q. (2023). Co-delivery of curcumin and quercetin in shellac nanocapsules for the synergistic antioxidant properties and cytotoxicity against colon cancer cells. Food Chem..

[66] Joshi T., Deepa P.R., Sharma P.K. (2022). Effect of different proportions of phenolics on antioxidant potential: Pointers for bioactive Synergy/Antagonism in foods and nutraceuticals. Proc. Natl. Acad. Sci. India Sect. B Biol. Sci..

[67] Silva F., Veiga F., Cardoso C., Dias F., Cerqueira F., Medeiros R., Paiva-Santos A.C. (2024). A rapid and simplified DPPH assay for analysis of antioxidant interactions in binary combinations. Microchem. J..

[68] Tang L., Liu R., Xu Y., Zhang X., Liu R., Chang M., Wang X. (2022). Synergistic and antagonistic interactions of α-tocopherol, γ-oryzanol and phytosterol in refined coconut oil. LWT.

[69] Liu R., Xu Y., Chang M., Tang L., Lu M., Liu R., Jin Q., Wang X. (2021). Antioxidant interaction of α-tocopherol, γ-oryzanol and phytosterol in rice bran oil. Food Chem..

[70] Liu G., Zhu W., Li S., Zhou W., Zhang H., Wang J., Liu X., Liang L., Xu X. (2022). Antioxidant capacity and interaction of endogenous phenolic compounds from tea seed oil. Food Chem..

[71] Hidalgo M., Sánchez-Moreno C., de Pascual-Teresa S. (2010). Flavonoid–flavonoid interaction and its effect on their antioxidant activity. Food Chem..

[72] Kurin E., Mučaji P., Nagy M. (2012). In vitro antioxidant activities of three red wine polyphenols and their mixtures: An interaction study. Molecules.

[73] Becker E.M., Ntouma G., Skibsted L.H. (2007). Synergism and antagonism between quercetin and other chain-breaking antioxidants in lipid systems of increasing structural organisation. Food Chem..

[74] Guo Y., Baschieri A., Amorati R., Valgimigli L. (2021). Synergic antioxidant activity of γ-terpinene with phenols and polyphenols enabled by hydroperoxyl radicals. Food Chem..

[75] Liang Z., Han D., Han F., Wu Z., Zhao X., Fu W., Wang W., Han D., Niu L. (2021). Novel strategy of natural antioxidant nutrition quality evaluation in food: Oxidation resistance mechanism and synergistic effects investigation. Food Chem..

[76] Ohta K., Motohira Y., Maruishi Y., Minami I., Kobayashi H., Ishikawa H. (2023). The combined effects of α-tocopherol and rosmarinic acid on the off-odor formation from lipid oxidation. Food Sci. Technol. Res..

[77] Zhang Q., Pan J., Liu H., Jiao Z. (2023). Characterization of the synergistic antioxidant activity of epigallocatechin gallate (EGCG) and kaempferol. Molecules.

[78] Kut K., Sitarz O., Kapusta I., Bartosz G., Sadowska-Bartosz I. (2025). Interaction of red cabbage extract with exogenous antioxidants. Int. J. Mol. Sci..

[79] Sitarz O., Bartosz G., Sadowska-Bartosz I. (2026). Interaction of red cabbage extract with exogenous antioxidants in ORAC assay. Int. J. Mol. Sci..

[80] Furdak P., Kut K., Bartosz G., Sadowska-Bartosz I. (2025). Comparison of various assays of antioxidant activity/capacity: Limited significance of redox potentials of oxidants/indicators. Int. J. Mol. Sci..

[81] Heo H.J., Kim Y.J., Chung D., Kim D.O. (2007). Antioxidant capacities of individual and combined phenolics in a model system. Food Chem..

[82] Rossetto M., Vanzani P., Mattivi F., Lunelli M., Scarpa M., Rigo A. (2002). Synergistic antioxidant effect of catechin and malvidin 3-glucoside on free radical-initiated peroxidation of linoleic acid in micelles. Arch. Biochem. Biophys..

[83] de Guzman R., Tang H., Salley S., Ng K.S. (2009). Synergistic effects of antioxidants on the oxidative stability of soybean oil-and poultry fat-based biodiesel. J. Am. Oil Chem. Soc..

[84] Galano A. (2025). Antioxidant activity at the molecular level: Exploring ways of action and computational tools to investigate them. Chem. Sci..

[85] Gulcin İ. (2025). Antioxidants: A comprehensive review. Arch. Toxicol..

[86] Baschieri A., Jin Z., Todtmann G., DiLabio G.A., Amorati R. (2025). Solvent effects on C–H abstraction by hydroperoxyl radicals: Implication for antioxidant strategies. J. Org. Chem..

[87] Suhag R., Angeli L., Scampicchio M., Ferrentino G. (2025). From quantity to reactivity: Advancing kinetic-based antioxidant testing methods for natural compounds and food applications. Compr. Rev. Food Sci. Food Saf..

[88] Wen N., Li M., Huo Y., Zhou Y., Ma Y., Gu Q., He M. (2025). Homogeneous and heterogeneous reaction mechanisms of synthetic phenolic antioxidants and their environmental effects. J. Environ. Chem. Eng..

[89] Frankel E.N., Huang S.W., Kanner J., German J.B. (1994). Interfacial phenomena in the evaluation of antioxidants: Bulk oils vs emulsions. J. Agric. Food Chem..

[90] Dar A.A., Bravo-Diaz C., Nazir N., Romsted L.S. (2017). Chemical kinetic and chemical trapping methods: Unique approaches for determining respectively the antioxidant distributions and interfacial molarities of water, counter-anions, and other weakly basic nucleophiles in association colloids. Curr. Opin. Colloid Interface Sci..

[91] Fang J.G., Lu M., Chen Z.H., Zhu H.H., Li Y., Yang L., Wu L.M., Liu Z.L. (2002). Antioxidant effects of resveratrol and its analogues against the free-radical-induced peroxidation of linoleic acid in micelles. Chem. Eur. J..

[92] Fang J.G., Zhou B. (2008). Structure–activity relationship and mechanism of the tocopherol-regenerating activity of resveratrol and its analogues. J. Agric. Food Chem..

[93] Sadowska-Bartosz I., Bartosz G. (2022). Evaluation of the antioxidant capacity of food products: Methods, applications and limitations. Processes.

[94] Ilyasov I.R., Beloborodov V.L., Selivanova I.A., Terekhov R.P. (2020). ABTS/PP decolorization assay of antioxidant capacity reaction pathways. Int. J. Mol. Sci..

[95] Schaich K.M., Tian X., Xie J. (2015). Hurdles and pitfalls in measuring antioxidant efficacy: A critical evaluation of ABTS, DPPH, and ORAC assays. J. Funct. Foods.

[96] Packer J.E., Slater T.F., Willson R.L. (1979). Direct observation of a free radical interaction between vitamin E and vitamin C. Nature.

[97] Niki E., Saito T., Kawakami A., Kamiya Y. (1984). Inhibition of oxidation of methyl linoleate in solution by vitamin E and vitamin C. J. Biol. Chem..

[98] Buettner G.R. (1993). The pecking order of free radicals and antioxidants: Lipid peroxidation, α-tocopherol, and ascorbate. Arch. Bioch. Biophys..

[99] DiLabio G.A., Wright J.S. (2000). Hemiketal formation of dehydroascorbic acid drives ascorbyl radical anion disproportionation. Free Radic. Biol. Med..

[100] Fukuzawa K., Ikebata W., Sohmi K. (1993). Location, antioxidant and recycling dynamics of alpha-tocopherol in liposome membranes. J. Nutr. Sci. Vitaminol..

[101] Barouh N., Bourlieu-Lacanal C., Figueroa-Espinoza M.C., Durand E., Villeneuve P. (2022). Tocopherols as antioxidants in lipid-based systems: The combination of chemical and physicochemical interactions determines their efficiency. Compr. Rev. Food Sci. Food Saf..

[102] Kadoma Y., Ishihara M., Okada N., Fujisawa S. (2006). Free radical interaction between vitamin E (alpha-, beta-, gamma-and delta-tocopherol), ascorbate and flavonoids. In Vivo.

[103] Pazos M., Andersen M.L., Medina I., Skibsted L.H. (2007). Efficiency of natural phenolic compounds regenerating α-tocopherol from α-tocopheroxyl radical. J. Agric. Food Chem..

[104] Mollica F., Gelabert I., Amorati R. (2022). Synergic antioxidant effects of the essential oil component γ-terpinene on high-temperature oil oxidation. ACS Food Sci. Technol..

[105] Amorati R., Ferroni F., Lucarini M., Pedulli G.F., Valgimigli L. (2002). A quantitative approach to the recycling of α-tocopherol by coantioxidants. J. Org. Chem..

[106] Tadolini B., Juliano C., Piu L., Franconi F., Cabrini L. (2000). Resveratrol inhibition of lipid peroxidation. Free Radic. Res..

[107] Foti M.C., Ingold K.U. (2003). Mechanism of inhibition of lipid peroxidation by γ-terpinene, an unusual and potentially useful hydrocarbon antioxidant. J. Agric. Food Chem..

[108] Grzesik M., Bartosz G., Dziedzic A., Narog D., Namiesnik J., Sadowska-Bartosz I. (2018). Antioxidant properties of ferrous flavanol mixtures. Food Chem..

[109] De Souza R.F., De Giovani W.F. (2004). Antioxidant properties of complexes of flavonoids with metal ions. Redox Rep..

[110] Bolocan N., Spinu O., Povar I. (2026). Commentary: Molecular mechanisms of true and apparent antioxidant synergism in foods. Food Chem. Mol. Sci..

[111] Kurechi T., Aizawa M., Kunugi A. (1983). Studies on the antioxidants XVIII: Oxidation product of tertiary butyl hydroquinone (TBHQ)(I). J. Am. Oil Chem. Soc..

[112] Cheng J., Li Y., Wang Y., Ye X., Wang H., Sun Z., Lu Z., Wei Y., Xu X. (2026). Kinetic mechanism of hydrogen atom transfer between polycyclic aromatic Quinones and glutathione. J. Photochem. Photobiol. A Chem..

[113] Tan D.X., Manchester L.C., Reiter R.J., Qi W.B., Karbownik M., Calvo J.R. (2000). Significance of melatonin in antioxidative defense system: Reactions and products. Neurosignals.

[114] Rosen J., Than N.N., Koch D., Poeggeler B., Laatsch H., Hardeland R. (2006). Interactions of melatonin and its metabolites with the ABTS cation radical: Extension of the radical scavenger cascade and formation of a novel class of oxidation products, C2-substituted 3-indolinones. J. Pineal Res..

[115] Arts M.J., Haenen G.R., Voss H.P., Bast A. (2004). Antioxidant capacity of reaction products limits the applicability of the Trolox Equivalent Antioxidant Capacity (TEAC) assay. Food Chem. Toxicol..

[116] Shalaby E.A., Mahmoud G.I., Shanab S.M. (2016). Suggested mechanism for the effect of sweeteners on radical scavenging activity of phenolic compounds in black and green tea. Front. Life Sci..

[117] Amorati R., Valgimigli L. (2012). Modulation of the antioxidant activity of phenols by non-covalent interactions. Org. Biomol. Chem..

[118] Spiegel M. (2022). Current trends in computational quantum chemistry studies on antioxidant radical scavenging activity. J. Chem. Inf. Model..

[119] Xiang Y., Xiang M., Mao Y., Huang L., He Q., Dong Y. (2025). Insights into structure-antioxidant activity relationships of polyphenol-phospholipid complexes: The effect of hydrogen bonds formed by phenolic hydroxyl groups. Food Chem..

[120] Gutteridge J.M. (1987). A method for removal of trace iron contamination from biological buffers. FEBS Lett..

[121] Welch K.D., Davis T.Z., Aust S.D. (2002). Iron autoxidation and free radical generation: Effects of buffers, ligands, and chelators. Arch. Biochem. Biophys..

[122] Bandyopadhyay P., Ghosh A.K., Ghosh C. (2012). Recent develpments on polyphenol–protein interactions: Effects on tea and coffee taste, antioxidant properties and the digestive system. Food Funct..

[123] Nedić O., Penezić A., Minić S., Radomirović M., Nikolić M., Ćirković Veličković T., Gligorijević N. (2023). Food antioxidants and their interaction with human proteins. Antioxidants.

[124] Sun X., Sarteshnizi R.A., Udenigwe C.C. (2022). Recent advances in protein–polyphenol interactions focusing on structural properties related to antioxidant activities. Curr. Opin. Food Sci..

[125] de Morais F.P., Pessato T.B., Rodrigues E., Mallmann L.P., Mariutti L.R., Netto F.M. (2020). Whey protein and phenolic compound complexation: Effects on antioxidant capacity before and after in vitro digestion. Food Res. Int..

[126] Jiang J., Zhang Z., Zhao J., Liu Y. (2018). The effect of non-covalent interaction of chlorogenic acid with whey protein and casein on physicochemical and radical-scavenging activity of in vitro protein digests. Food Chem..

[127] Feng Y., Jin C., Lv S., Zhang H., Ren F., Wang J. (2023). Molecular mechanisms and applications of polyphenol-protein complexes with antioxidant properties: A review. Antioxidants.

[128] Günal-Köroğlu D., Ozkan G., Kamiloglu S., Tomas M., Ozdal T., Capanoglu E. (2026). Digestion of protein-phenolic complex: Interactions, matrix effects and digestibility. Crit. Rev. Food Sci. Nutr..

[129] Huynh H.D., Thi T.H.T., Thi T.X.T., Nargotra P., Wang H.M.D., Liu Y.C., Kuo C.H. (2026). Recent insights into protein-polyphenol complexes: Molecular mechanisms, processing technologies, synergistic bioactivities, and food applications. Molecules.

[130] Shi W., Xie H., Ouyang K., Wang S., Xiong H., Woo M.W., Zhao Q. (2024). The effect of rice protein-polyphenols covalent and non-covalent interactions on the structure, functionality and in vitro digestion properties of rice protein. Food Chem..

[131] Lamuela-Raventós R.M., Apak R., Capanoglu E., Shahidi F. (2018). Folin–Ciocalteu method for the measurement of total phenolic content and antioxidant capacity. Measurement of Antioxidant Activity & Capacity: Recent Trends and Applications.

[132] Pérez M., Dominguez-López I., Lamuela-Raventós R.M. (2023). The chemistry behind the Folín–Ciocalteu method for the estimation of (poly) phenol content in food: Total phenolic intake in a Mediterranean dietary pattern. J. Agric. Food Chem..

[133] Hossain M.B., Ahmed L., Martin-Diana A.B., Brunton N.P., Barry-Ryan C. (2023). Individual and combined antioxidant activity of spices and spice phenolics. Antioxidants.

[134] Joshi T., Agrawal K., Mangal M., Deepa P.R., Sharma P.K. (2024). Measurement of antioxidant synergy between phenolic bioactives in traditional food combinations (legume/non-legume/fruit) of (semi) arid regions: Insights into the development of sustainable functional foods. Discov. Food.

[135] Uduwana S., Abeynayake N., Wickramasinghe I. (2023). Synergistic, antagonistic, and additive effects on the resultant antioxidant activity in infusions of green tea with bee honey and *Citrus limonum* extract as additives. J. Agric. Food Res..

[136] Stunda-Zujeva A., Berele M., Lece A., Šķesters A. (2023). Comparison of antioxidant activity in various Spirulina containing products and factors affecting it. Sci. Rep..

[137] Liu X., He F., Zhao L., Zhou A., Liu X. (2014). Synergistic antioxidant activity of sweet potato extracts in combination with tea polyphenols and Pueraria flavonols In Vitro. Adv. J. Food Sci. Technol..

[138] Putra I.M.W.A., Fakhrudin N., Kusumawati I.G.A.W., Nurrochmad A., Wahyuono S. (2022). Antioxidant properties of extract combination of *Coccinia grandis* and *Blumea balsamifera*: An in vitro synergistic effect. J. Herbmed Pharmacol..

[139] Putra I.M.W.A., Suarjana N. (2025). Evaluation of phosphomolybdenum and ferric reducing antioxidant power assays in extract combinations of *Mangifera indica* and *Euphorbia hirta*. Walisongo J. Chem..

[140] Yap V.L., Tan L.F., Rajagopal M., Wiart C., Selvaraja M., Leong M.Y., Tan P.L. (2023). Evaluation of phytochemicals and antioxidant potential of a new polyherbal formulation TC-16: Additive, synergistic or antagonistic?. BMC Complement. Med. Ther..

[141] Yakoubi R., Megateli S., Sadok T.H., Bensouici C., Bağci E. (2021). A synergistic interactions of Algerian essential oils of *Laurus nobilis* L.; *Lavandula stoechas* L. and *Mentha pulegium* L. on anticholinesterase and antioxidant activities. Biocatal. Agric. Biotechnol..

[142] Benamar-Aissa B., Gourine N., Ouinten M., Harrat M., Benarfa A., Yousfi M. (2023). Synergistic effects of essential oils and phenolic extracts on antioxidant activities responses using two *Artemisia* species (*A. campestris* and *A. herba alba*) combined with *Citrus aurantium*. Biocatal. Agric. Biotechnol..

[143] Serpen A., Gökmen V., Fogliano V. (2012). Total antioxidant capacities of raw and cooked meats. Meat Sci..

[144] Cömert E.D., Gökmen V. (2022). Effect of food combinations and their co-digestion on total antioxidant capacity under simulated gastrointestinal conditions. Curr. Res. Food Sci..

[145] Bamidele O.P., Moyo B., Madala N.E., Ramashia S.E. (2025). Chemical properties, antioxidant activities and metabolic profile of 810 mixed blackjack and jute vegetables. Food Chem..

[146] Close D.C., McArthur C. (2002). Rethinking the role of many plant phenolics–protection from photodamage not herbivores?. Oikos.

[147] Šamec D., Karalija E., Šola I., Vujčić Bok V., Salopek-Sondi B. (2021). The role of polyphenols in abiotic stress response: The influence of molecular structure. Plants.

[148] Moctezuma C., Hammerbacher A., Heil M., Gershenzon J., Méndez-Alonzo R., Oyama K. (2014). Specific polyphenols and tannins are associated with defense against insect herbivores in the tropical oak *Quercus oleoides*. J. Chem. Ecol..

[149] Singh S., Kaur I., Kariyat R. (2021). The multifunctional roles of polyphenols in plant-herbivore interactions. Int. J. Mol. Sci..

[150] Stiller A., Garrison K., Gurdyumov K., Kenner J., Yasmin F., Yates P., Song B.H. (2021). From fighting critters to saving lives: Polyphenols in plant defense and human health. Int. J. Mol. Sci..

[151] Daglia M. (2012). Polyphenols as antimicrobial agents. Curr. Opin. Biotechnol..

[152] Mandal M.K., Domb A.J. (2024). Antimicrobial activities of natural bioactive polyphenols. Pharmaceutics.

[153] Ross J.A., Kasum C.M. (2002). Dietary flavonoids: Bioavailability, metabolic effects, and safety. Ann. Rev. Nutr..

[154] Karakaya S. (2004). Bioavailability of phenolic compounds. Crit. Rev. Food Sci. Nutr..

[155] Chen L., Cao H., Huang Q., Xiao J., Teng H. (2022). Absorption, metabolism and bioavailability of flavonoids: A review. Crit. Rev. Food Sci. Nutr..

[156] Forman H.J., Davies K.J., Ursini F. (2014). How do nutritional antioxidants really work: Nucleophilic tone and para-hormesis versus free radical scavenging In Vivo. Free Radic. Biol. Med..

[157] Lotito S.B., Zhang W.J., Yang C.S., Crozier A., Frei B. (2011). Metabolic conversion of dietary flavonoids alters their anti-inflammatory and antioxidant properties. Free Radic. Biol. Med..

[158] Sarkar C., Chaudhary P., Jamaddar S., Janmeda P., Mondal M., Mubarak M.S., Islam M.T. (2022). Redox activity of flavonoids: Impact on human health, therapeutics, and chemical safety. Chem. Res. Toxicol..

[159] Fraga C.G., Oteiza P.I. (2011). Dietary flavonoids: Role of (−)-epicatechin and related procyanidins in cell signaling. Free Radic. Biol. Med..

[160] Sun C., Zhang J., Hou J., Hui M., Qi H., Lei T., Zhang X., Zhao L., Du H. (2023). Induction of autophagy via the PI3K/Akt/mTOR signaling pathway by Pueraria flavonoids improves non-alcoholic fatty liver disease in obese mice. Biomed. Pharmacother..

[161] Mazurakova A., Koklesova L., Csizmár S.H., Samec M., Brockmueller A., Šudomová M., Biringer K., Kudela E., Pec M., Samuel S.M. (2024). Significance of flavonoids targeting PI3K/Akt/HIF-1α signaling pathway in therapy-resistant cancer cells—A potential contribution to the predictive, preventive, and personalized medicine. J. Adv. Res..

[162] Siddiqui S., Abdelwadoud M.E., Vyas M., Saeed M., Mazumder A., Saeed A. (2023). Targeting Wnt/β-catenin pathway by flavonoids: Implication for cancer therapeutics. Nutrients.

[163] Wen J., Qin S., Li Y., Zhang P., Zhan X., Fang M., Shi C., Mu W., Kan W., Zhao J. (2023). Flavonoids derived from licorice suppress LPS-induced acute lung injury in mice by inhibiting the cGAS-STING signaling pathway. Food Chem. Toxicol..

[164] Tarahovsky Y.S., Muzafarov E.N., Kim Y.A. (2008). Rafts making and rafts braking: How plant flavonoids may control membrane heterogeneity. Mol. Cell. Biochem..

[165] Ding M., Zhu Y., Xu X., He H., Jiang T., Mo X., Wang Z., Yu W., Ou H. (2024). Naringenin inhibits acid sphingomyelinase-mediated membrane raft clustering to reduce NADPH oxidase activation and vascular inflammation. J. Agric. Food Chem..

[166] Duhon D., Bigelow R.L.H., Coleman D.T., Steffan J.J., Yu C., Langston W., Kevil C.G., Cardelli J.A. (2010). The polyphenol epigallocatechin-3-gallate affects lipid rafts to block activation of the c-Met receptor in prostate cancer cells. Mol. Carcinog..

[167] Zhang M., Zhang G., Meng X., Wang X., Xie J., Wang S., Wang B., Wang J., Liu S., Huang Q. (2024). Reduction of the oxidative damage to H2O2-induced HepG2 cells via the Nrf2 signalling pathway by plant flavonoids Quercetin and Hyperoside. Food Sci. Hum. Wellness.

[168] Moratilla-Rivera I., Sánchez M., Valdés-González J.A., Gómez-Serranillos M.P. (2023). Natural products as modulators of Nrf2 signaling pathway in neuroprotection. Int. J. Mol. Sci..

[169] Zhao R., Bai W., Tian Z., Li X., Sun R., Wang Z., Yan X., Jia J. (2025). Cistanche flavonoids activate the Keap1-Nrf2-ARE signaling pathway in improving cognitive dysfunction in Alzheimer’s disease: A review. J. Alzheimers Dis..

[170] Gote S., Dubey S., Nargund S.L., Thapa S. (2025). A systematic review of natural products targeting Nrf2-Keap1-ARE pathway and their influence on neurodegenerative disorders. Inflammopharmacology.

[171] Rasouli H., Farzaei M.H., Khodarahmi R. (2017). Polyphenols and their benefits: A review. Int. J. Food Prop..

[172] Leri M., Scuto M., Ontario M.L., Calabrese V., Calabrese E.J., Bucciantini M., Stefani M. (2020). Healthy effects of plant polyphenols: Molecular mechanisms. Int. J. Mol. Sci..

